# The methanogen core and pangenome: conservation and variability across biology’s growth temperature extremes

**DOI:** 10.1093/dnares/dsac048

**Published:** 2022-12-01

**Authors:** Paula Prondzinsky, Sakae Toyoda, Shawn Erin McGlynn

**Affiliations:** Earth-Life Science Institute, Tokyo Institute of Technology, 2-12-1 Ookayama, Meguro-ku, 152-8550 Tokyo, Japan; Department of Chemical Science and Engineering, Tokyo Institute of Technology, 4259 Nagatsuta-cho, Midori-ku, 226-8503 Yokohama, Japan; Department of Chemical Science and Engineering, Tokyo Institute of Technology, 4259 Nagatsuta-cho, Midori-ku, 226-8503 Yokohama, Japan; Earth-Life Science Institute, Tokyo Institute of Technology, 2-12-1 Ookayama, Meguro-ku, 152-8550 Tokyo, Japan; Center for Sustainable Resource Science, RIKEN, 2-1 Hirosawa, Wako, 351-0198 Saitama, Japan; Blue Marble Space Institute of Science, Seattle, WA 98154, USA

**Keywords:** methanogenesis, genome comparison, temperature adaptations, pangenome, physiology

## Abstract

Temperature is a key variable in biological processes. However, a complete understanding of biological temperature adaptation is lacking, in part because of the unique constraints among different evolutionary lineages and physiological groups. Here we compared the genomes of cultivated psychrotolerant and thermotolerant methanogens, which are physiologically related and span growth temperatures from −2.5°C to 122°C. Despite being phylogenetically distributed amongst three phyla in the archaea, the genomic core of cultivated methanogens comprises about one-third of a given genome, while the genome fraction shared by any two organisms decreases with increasing phylogenetic distance between them. Increased methanogenic growth temperature is associated with reduced genome size, and thermotolerant organisms—which are distributed across the archaeal tree—have larger core genome fractions, suggesting that genome size is governed by temperature rather than phylogeny. Thermotolerant methanogens are enriched in metal and other transporters, and psychrotolerant methanogens are enriched in proteins related to structure and motility. Observed amino acid compositional differences between temperature groups include proteome charge, polarity and unfolding entropy. Our results suggest that in the methanogens, shared physiology maintains a large, conserved genomic core even across large phylogenetic distances and biology’s temperature extremes.

## Introduction

Life is a chemical process: it follows that temperature is a key determinant both of rate and reaction favourability.^1,2^ Temperature effects can be seen across biology,^3^ and it is of interest to consider these from an evolutionary perspective. Although recent phylogenetic trees do not necessarily indicate a basal placement of thermophiles, the history of life in relation to temperature has been debated ever since the first trees placed thermophilic lineages in basal positions^4–7^ suggesting that taxonomic evolution may be entangled with temperature.

An ongoing challenge has been to identify how organisms adapt to life at high temperatures.^16–21^ Able to grow from −2.5 to 122°C,^8,9^ methanogens span perhaps the largest temperature range of any single physiological group. Methanogens generally produce methane from CO_2_ and H_2_, acetate and/or methylated compounds,^10,11^ and are found in environments including animal rumens,^12^ to wetlands,^13^ to hydrothermal vents,^14^ to subglacial sediments.^15^

To avoid confounding factors when considering different lifestyles and phylogenetic backgrounds, Lecocq et al.^22^ investigated the Methanococcales, finding that amino acid substitution patterns, rather than the acquisition of orthogroups best explain thermotolerance. Other previous comparative genomic studies of methanogens focused on pairs of species or organisms within a specific clade.^23–25^

We sought to investigate possible genome adaptations to temperature by comparatively analysing genomes of cultured organisms for which growth temperature and substrate utilization information is available. By analysing methanogens as a physiologically similar yet phylogenetically diverse group, we specifically aimed to constrain metabolic variables and to search for temperature effects on genome evolution which might exist as general adaptation strategies rather than as synapomorphies unique to specific phylogenetic groups. Our study compliments and adds to our understandings of what makes a methanogen a methanogen, and how they have genomically evolved over time.

## Methodology

### Establishing a methanogen database

As a first step, the diversity of alkane-activating archaea was investigated based on the presence of alpha, beta and gamma subunits of the methyl-coenzyme M reductase (Mcr) enzyme using Annotree v1.2.0.^26^ The KEGG UniRef100 database^27^ was used to query the alpha (K00399), beta (K00401) and gamma (K00402) subunits in archaea with default parameters (min. 30% identity, max. E value 0.00001, min. 70% subject and query alignment). This search resulted in a total of 290 genome hits across six archaeal phyla including Halobacteriota (156), Methanobacteriota (94), Thermoplasmatota (27), Thermoproteota (10), Asgardarchaeota (2) and Hadarchaeota (1). Their protein sequences were downloaded from the GTDB data repository (GTDB release 95.^28^ A total of 255 genomes were considered for further analyses after a quality cut-off (checkM score ≥90). In previous taxonomic descriptions, methanogens were separated into Class one (Methanobacteriota) and Class two (Halobacteriota) methanogens within the Euryarchaeota phylum,^29^ however here we describe their taxonomy using that of GTDB release 95.

Temperature data for cultured organisms were accessed through the Database of Growth TEMPeratures of Usual and Rare Prokaryotes (TEMPURA, Sato et al.^30^) and substrate data was added from the PhyMet2 database.^31^ This data compilation is available in Supplementary Table S1. Phylogenetic trees with annotations were visualized using the interactive tree of life online tool.^32^

A total of 86 organisms had temperature data available and were used for the main analyses herein. There is no universal definition for growth temperatures to classify organisms into psychrotolerant and thermotolerant groups. Here we defined these temperature groups based on the overall distribution of growth temperatures for methanogenesis (Supplementary Fig. S1). Organisms were classified into psychrotolerant organisms with a minimum growth temperature ≤15°C and with no growth observed above 45°C. Thermotolerant organisms have a maximum growth temperature ≥45°C and no growth observed below 15°C. Organisms spanning ≤15°C to ≥45°C are considered as mesophilic. This classification resulted in 21 psychrotolerant, 41 thermotolerant and 24 mesophilic species.

### Core and pangenomes

Core- and pangenomes were established with OrthoFinder^33^ running with default parameters on both the 255 species with checkM score ≥ 90 and the 86 species with available temperature data. The resulting orthogroups were separated into the core (here we used the extended core of orthogroups present in more than 95% of organisms to account for incomplete genomes), shared (orthogroups present in at least two but less than 95% of organisms) and unique (genes present only in one organism) groups. These groupings were used for all subsequent analyses.

For comparison to other microbes, genera with temperature data for at least five of their member species were selected from the TEMPURA database and five species selected randomly for core genome analyses using Proteinortho,^34^ run with the default parameters. The number of species was kept constant to account for the reduction of the core size with the addition of species^35^ and to keep the analyses comparable. Fourteen archaeal and bacterial genera, including two methanogenic ones, are represented by at least five species and were used for this analysis, with eight thermotolerant (*Pyrococcus*, *Pyrobaculum*, *Thermococcus*, *Methanocaldococcus*, *Acidianus*, *Thermotoga*, *Caldicellulosiruptor*, and *Fervidobacterium*), four mesophilic (*Acholeplasma*, *Bacteroides*, *Eubacterium* and *Methanosarcina*) and two psychrotolerant (*Cryobacterium* and *Paraglaciecola*) genera.

### Composition and function

Functional annotations of all genes in each orthogroup were made with eggnogmapper version 5.^36^ Initial overviews of functional differences between orthogroup and temperature groups were done based on cluster of orthologous genes (COG) categories. Subsequently KEGG orthology output from eggnogmapper was used to map reactions unique to temperature groups as well as substrate groups with the KEGG pathway module.^27^

Amino acid biases were assessed by counting amino acids coded by open reading frames in the whole genomes as well as core and shared fractions. Based on amino acid compositions, proteome properties were calculated based on Gromiha et al.^37^ indicating the average property of a residue within a species’ proteome. To test for statistically significant trends with temperature in amino acid composition and amino acid residue properties, Pearson correlation *P*-values were calculated for amino acid counts as well as residue properties for each species using the statistical functions module in python’s scipy package^38^ and tested for statistical significance with the Benjamini Hochberg correction using the fdr_correction function with method = ‘indep’ in the mne module in python.^39^

### Ancestral reconstruction for orthogroups

Count^40^ was used for estimations of gene gain and loss events using Wagner parsimony as well as to establish rate model optimization using the gain–loss-duplication model^41^ with the Poisson family size distribution at the root. Lineage-specific variations were left unspecified, and all other parameters were kept at default. The input data for this analysis was the group of 86 methanogens associated with growth temperature data.

## Results and discussion

### Phylogenetic distribution of growth temperature and physiology among methanogens

Using Annotree, we found 290 archaeal genomes contain the Mcr alpha, beta and gamma subunits, out of which 255 are considered to have a high enough quality for analyses (checkM score ≥ 90). Those genomes range in size from 1.112 Mbp (WYZ-LMO11 sp004348015 of the *Methanomethyliaceae* family) to 5.752 Mbp (*Methanosarcina acetivorans*) and span five phyla, 17 classes, 21 orders, 38 families and 99 genera, which highlights the continued taxonomic expansion of Mcr containing archaea.^11,42,43^ A list of these genomes with information on their size, quality and where available growth temperature ranges, isolation environments and growth substrates of species is available in Supplementary Table S1.

**Figure 1.**
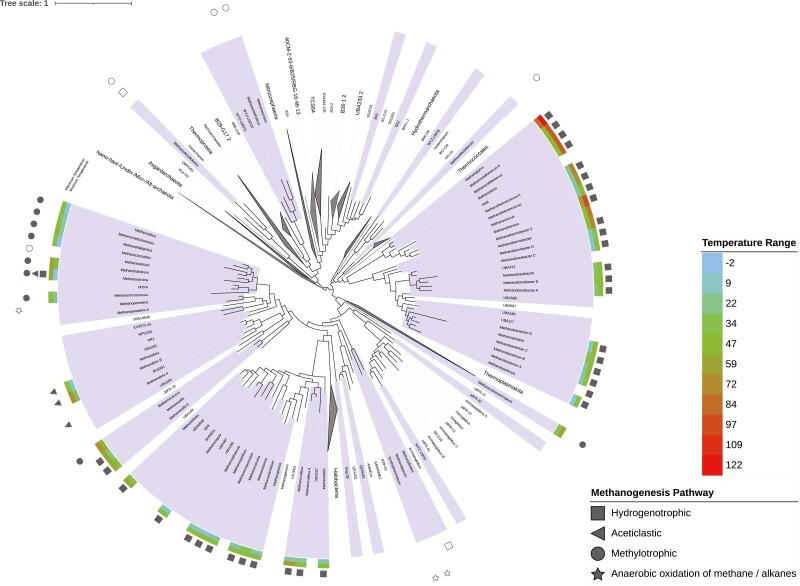
Genera containing mcrABG (marked in purple) throughout the archaeal tree of life. Coloured rings indicate minimum and maximum growth temperatures in °C observed within each genus, symbols indicate the substrate used for alkane metabolisms. Unfilled symbols indicate putative pathways which were inferred for yet uncultured species.

Among the 255 high-quality archaeal genomes, substrate data from cultivation studies is available for 112^31^ (Fig. 1). The *Methanomicrobia, Methanococci, Methanocellia* and *Methanobacteria* genera are strictly hydrogenotrophic, *Methanotrichales* strictly aceticlastic and all genera in the *Methanosarcinaceae* family besides *Methanosarcina* are strictly methylotrophic, while some species within *Methanosarcina* are capable of methanogenesis from CO_2_ and H_2_, acetate and/or methylated compounds. Eighty-three species have capabilities for hydrogenotrophic methanogenesis, while 72 are strict hydrogenotrophs unable to use other methanogenesis pathways. Psychrotolerant hydrogenotrophic organisms include members of the class Methanomicrobia, whereas thermotolerant ones include Methanopyri, Methanobacteria and Methanococci. With the exception of *Methermicoccus shengliensis* (methylotroph) and *Methanothrix B thermoacetophila* (aceticlastic), all methanogens with maximum growth temperatures ≥ 65°C are hydrogenotrophic. Thirty species are methylotrophic with a majority of these being psychrotolerant (Methanosarcinales) and 20 being strict methylotrophs. Finally, 17 are aceticlastic, mostly psychrotolerant species (Methanotrichales), with seven strict aceticlastic organisms. Other growth substrates for methanogenesis include butanol, 2-butanol, isobutanol, propanol, 2-propanol, carbon monoxide, cyclopentanol, dimethyl sulfide, ethanol, and formate, though these were omitted from our analysis since these substrates have been less frequently tested for growth compared with hydrogen, acetate and methyl-compounds.^31^

**Figure 2.**
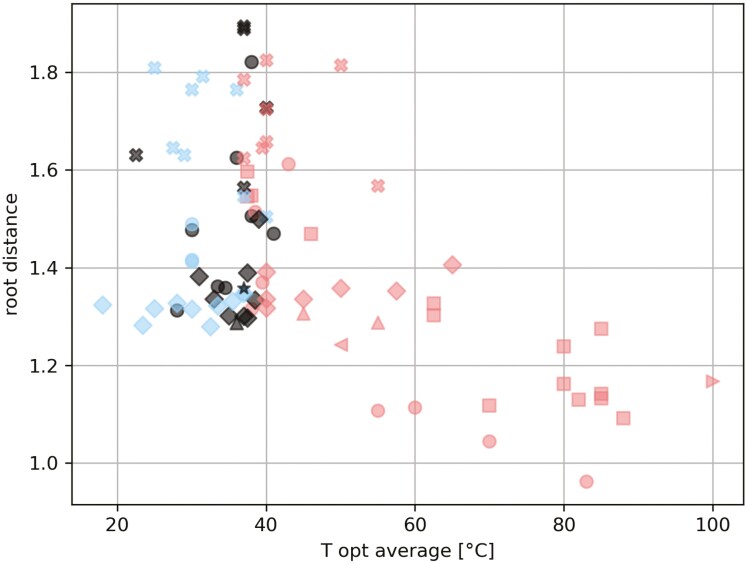
Branch lengths from the root of the archaeal tree to each species versus their optimal growth temperatures. Colours indicate temperature groups used in this study (blue—psychrotolerant, red—thermotolerant, black—no group/mesophilic. Shapes indicate class level taxonomy (circle—Methanobacteria, upward triangle—Methanocellia, square—Methanococci, x—Methanomicrobia, left point triangle—Methanonatronarchaeia, right point triangle—Methanopyri, diamond—Methanosarcinia, star—Thermoplasmata).

Thermotolerant organisms are associated with the shortest branches to the root of the archaeal species tree (Fig. 2; this figure is also redrawn using minimum and maximum growth temperatures in Supplementary Fig. S2). This follows the initial observation of Woese,^44^ who found that hyperthermophilic archaea were basal in the tree of life. In the case of the methanogens, the combined observation of short branch lengths and small genomes (see below, Fig. 4A) may run counter to a thermo-reduction hypothesis of genome evolution.^45^

### The methanogen pan- and core genomes

Eighty-six isolated methanogens have growth temperature data available in the TEMPURA database^30^ (a species tree is shown in Supplementary Fig. S4). These span 3 phyla, 8 classes, 10 orders, 19 families and 45 genera. A total of 211,659 genes from these 86 organisms were grouped into 10,131 orthogroups, out of which 791 orthogroups containing 2,329 genes are unique to a single genome. The core includes 330 orthogroups, which are shared by all 86 species, while 552 orthogroups belong to the extended core, which is shared by more than 95% of species. We adopt this relaxed measure proposed by Charlebois and Doolittle^35^ to account for partially incomplete genomes.

**Figure 3.**
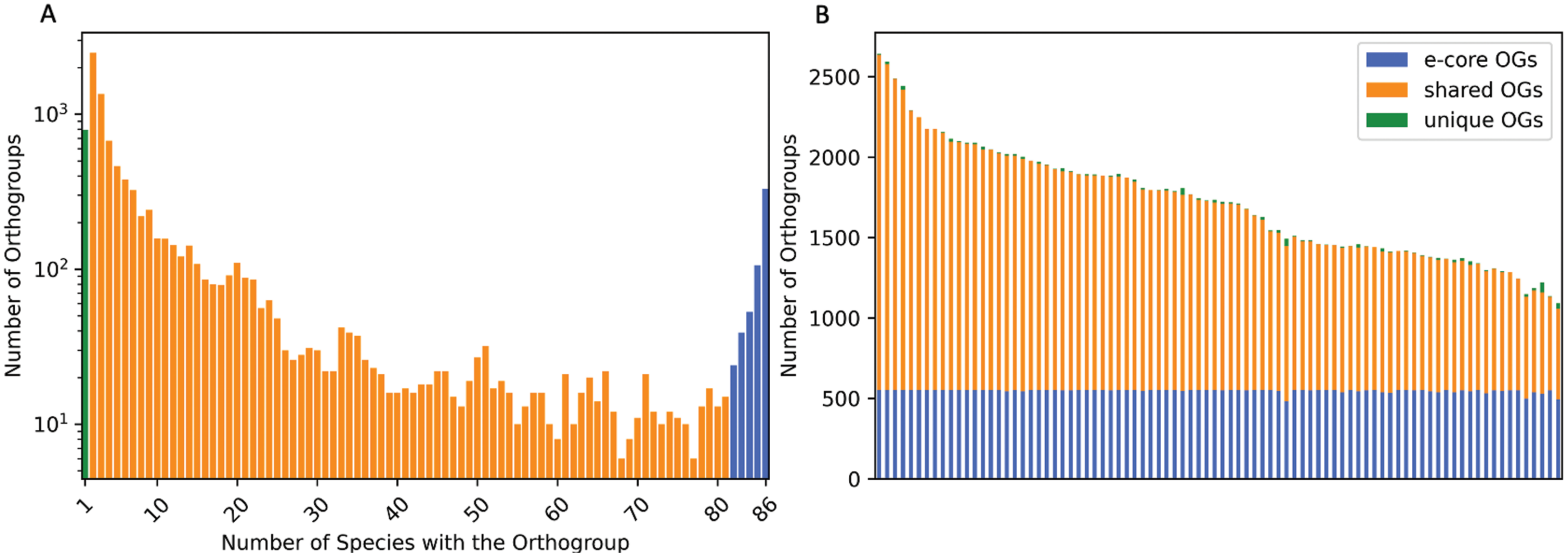
Core- and pangenome analysis of cultivated methanogens. (A) Distribution of the number of orthogroups across the methanogens. (B) Core- and pangenomes of the methanogens, where each bar is one species. Colours in both A and B indicate extended core (shared by at least 95% of the organisms), shared and unique genome fractions.

The extended core of these 86 methanogens consists largely of genes related to biosynthesis (57%), coenzyme transport and metabolism (13%). Eleven percent of genes have unknown functions and the remaining 19% are functionally related to lipid biosynthesis, ion transport, secondary metabolites, intracellular transports, motility, cell wall and membrane biogenesis, cell cycle control or carbohydrate transport (Supplementary Fig. S5). The remaining 8,788 orthogroups found in these species belong to the shared fraction of the pan-genome; those genes shared by at least two but less than 95% of organisms in the analysis. Distributions of the number of orthogroup sizes are shown in Fig. 3A. Extended core- and pan-genome sizes for each genome are shown in Fig. 3B.

The mean size of the extended genomic core is 33.74% (30% in psychrotolerant and 35% in thermotolerant organisms). This fraction is large, considering that these organisms span three different phyla and comparable core sizes can be found at the genus level (e.g. *Comamonas*^46^ or *Streptococcus*^47^). Interestingly, a large core fraction between phylogenetically diverse thermoacidophilic archaea was also observed.^48^ In that case, the constraints of life at low pH were thought to contribute to the large shared genome core. For the methanogens however, the environmental conditions are not constrained: in addition to temperature, growth pH of the organisms studied here ranges from 4.3 to 10,^31^ suggesting that the methanogen core genome may be constrained by the biochemical requirements of methanogenesis.

The addition of more genomes into core comparison analyses will reduce the core size.^35^ We find that when including all genomes that contain McrABG and have a checkM score of ≥90, the extended core contains 403 orthogroups, which is a 27% reduction in comparison to the core size observed when including only the 86 cultivated members. This set includes 255 genomes from methanogens, methanotrophs and other alkane-activating archaea: it appears to be the common core for alkane metabolizing archaea (all orthogroups for this analysis in Supplementary Table S2).

### Temperature and methanogen genome content

Thermal adaptation in the methanogens does not appear to be accomplished by the use of specific orthologous gene sets: There is neither a thermotolerant nor psychrotolerant specific core (i.e. orthogroups found in all organisms of one temperature group but not in the other). This being said, there are genes found exclusively in one temperature but not the other. Thermotolerant organisms are found with (metal) transporter genes and transcriptional regulators and contain the DNA repair gene rad51 and reverse gyrase, while psychrotolerant organisms have genes related to membrane and salt transport, membrane fusion, signaling and motility, chaperones and the DNA repair gene recN (comprehensive list in Table 1). Since these genes are not conserved in the temperature groups, they might be related to environmental factors other than temperature, such as high metal concentrations at hydrothermal vent systems, or high salinity in cold environments requiring additional membrane proteins and salt transporters in psychrotolerant organisms.

**Table 1.** Selected temperature group specific functional proteins

Psychrotolerant specific	COG category	Thermotolerant specific
VsrA DNA mismatch endonuclease	L	TopG, Rgy Reverse gyrase
RadN DNA repair	L	Rad51 DNA repair
YtrF Acetoin utilization transporter	V	EtaA Exfoliative toxin A/B
BlpB Membrane fusion protein	VM/M	ZipB, TC.BAT1, OpuD, BetL, BetT, BetS, ChaA, ChrA, PhaA Electrochemical potential driven transporters
WzxC Lipopolysaccharide transporter	M/P	AfuA, AfuB Iron transporters
RscC Capsule synthesis	M/P	MntA, MntB, MntC Manganese transporter
RfpC Cell-to-cell signaling	T/P	MtsA Iron/manganese/copper transporter
K20974 Swarming activity and biofilm formation	T/P	PsaA, ScaA, SloC Zinc/manganese transporter
HtpG Molecular chaperone	O/P	ABC.MN.A, ABC.MN.P, ABC.MN.S Manganese/iron transporter
WprA Cell wall associated protease	O/P	SLC39A9 Metal ion transporter
ChpA Chemosensory pili system protein	W/P	SLC42A Rh ammonium transporter
MshQ MSHA pilus biogenesis protein	W/P	FieF Ferrous iron efflux pump
SLC7A4 Cationic amino acid transporter/glycoprotein associated	E/J	SelB Selenocysteine specific elongation factor
YfeH Sodium bile salt cotransporter	-/U	CpaA, TadV, CpaE, TadZ Tight adherence export apparatus
	—	SLC24A3 Na^+^/Ca^2+^ − K^+^ exchanger

Selected proteins found in one, but not the other temperature group. COG categories: L: replication, recombination and repair, V: defense mechanism, M: cell Wall/membrane/envelope biogenesis, P: inorganic ion transport and metabolism, T: signal transduction mechanism, O: post-translational modification, protein turnover and chaperones, W: extracellular structures, E: amino acid transport and metabolism, J: translation, ribosomal structure and biogenesis, U: intracellular trafficking, secretion and vesicular transport. The slash mark in the COG column separates the COG category designation for the genes listed on the left or right.

It may be that reverse gyrase is the only conserved and specific hyperthermophile protein,^49,50^ while chaperones and genes involved in membrane and cell wall biogenesis have been implicated in psychrotolerant species.^51^ Our analysis suggests that thermal adaptation in the methanogens may be largely accomplished by mechanisms other than the use of specific genes—perhaps as detailed below by amino acid substitution patterns.

In a previous analysis with hyperthermophilic bacteria and archaea,^52^ 107 COGs specific to hyperthermophilic archaea were identified out of which 58 are shared across the domains. From these 58 COGs, those with known functions belong to predicted DNA repair systems, metabolic functions (*n* = 9), replication and repair genes (*n* = 4), and one each to translation-related genes and the COG category transcription-related genes and cellular processes. In our analysis, the majority of temperature group specific orthogroups have no functional assignment, and these may be experimental targets for understanding the molecular basis of thermotolerance.

### Temperature and methanogen genome size

**Figure 4.**
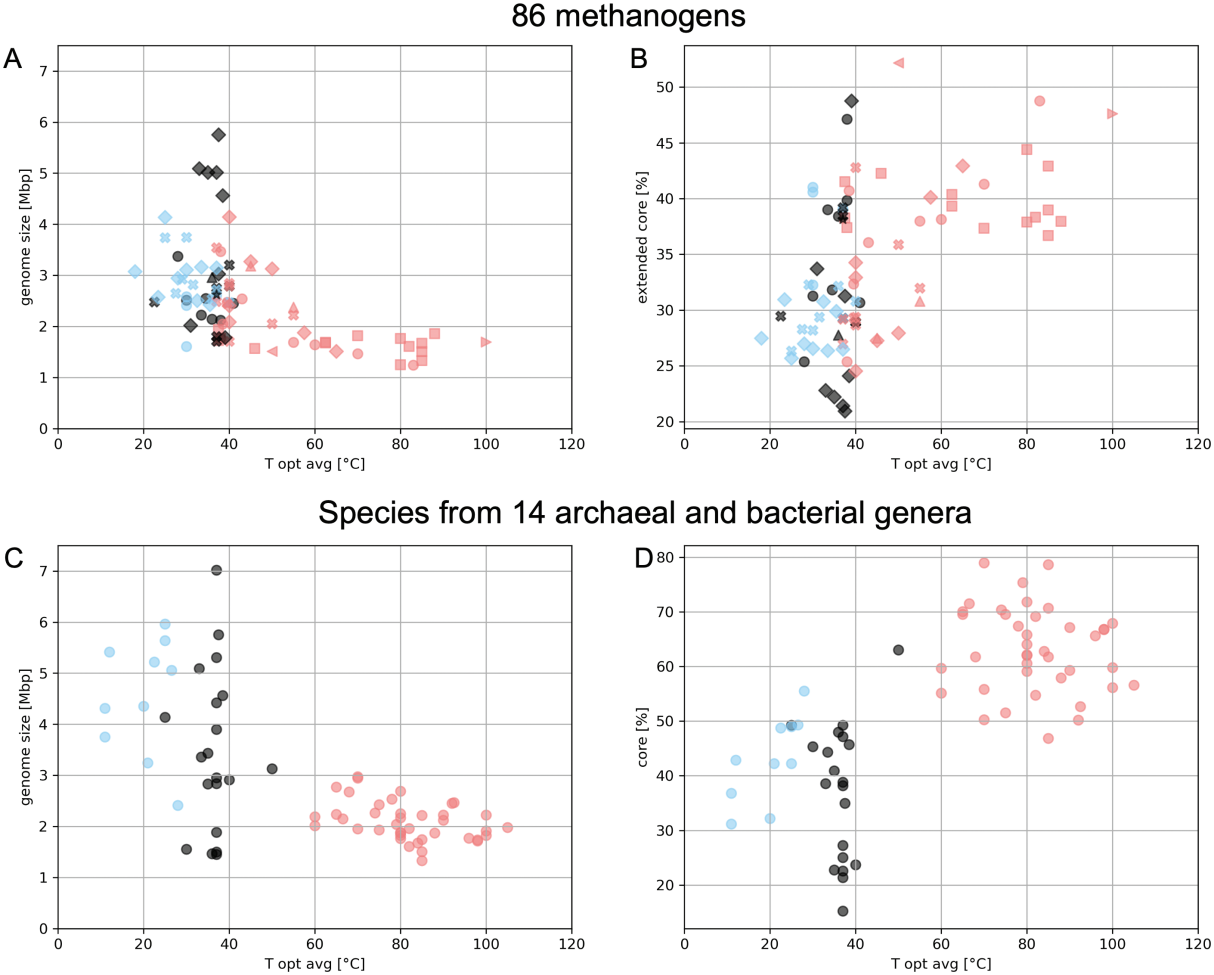
(A) Distribution of genome sizes with *T*_opt_ for 86 methanogen species. (B) Core sizes with *T*_opt_ for 86 methanogen species. (C) Distribution of genome sizes with *T*_opt_ for each species across the genera Pyrococcus, Pyrobaculum, Thermococcus, Methanocaldococcus, Acidianus, Thermotoga, Caldicellulosiruptor, Fervidobacterium, Acholeplasma, Bacteroides, Eubacterium, Methanosarcina, Cryobacterium and Paraglaciecola. Five genomes from each genus were randomly chosen so as to have equal genomic representation between the different groups. (D) Core sizes with *T*_opt_ for each species across the aforementioned genera. Colours in (A)—(D) indicate temperature groups used in this study (blue—psychrotolerant, red—thermotolerant, black—no group/mesophilic. Shapes in (A) and (B) indicate class level phylogenies (circle—Methanobacteria, upward triangle—Methanocellia, square—Methanococci, x—Methanomicrobia, left point triangle—Methanonatronarchaeia, right point triangle—Methanopyri, diamond—Methanosarcinia, star—Thermoplasmata).

The genomes of thermotolerant species are on average ~30% smaller than those of organisms growing at colder temperatures (Fig. 4A; also see Supplementary Fig. S3 for a comparison of genome size with minimum and maximum growth temperature), a trend that is not unique to methanogens and has been observed throughout the bacteria and archaea;^30^Fig. 4C). Among all methanogens, genome size ranges from 1.24 Mbp (*Methanothermus fervidus*) to 5.75 Mbp (*Methanosarcina acetivorans*). For psychrotolerant species the mean genome size is 2.85 Mbp with 2748 ORFs (min. 1.61 Mbp in *Methanobrevibacter D filiformis*, max. 4.14 Mbp in *Methanosarcina lacustris*), while for their thermotolerant counterparts the mean genome size is 2.12 Mbp with 2096 ORFs (min. 1.24 Mbp in *Methanothermus fervidus*, max. 4.14 Mbp in *Methanosarcina mazei*).

To investigate if core size is correlated with growth temperature, the 86 methanogens and 5 species each from a total of 14 bacterial and archaeal genera were plotted. As temperature increases, genome size decreases while the core fraction increases both for the methanogens and archaea and bacteria (Fig. 4B and D). The two methanogenic genera included in these analyses (*Methanocaldococcus* and *Methanosarcina*, both containing more than five species) group with other genera of similar growth temperature ranges (Fig. 4D).

### Substrate-specific orthogroups

Between the substrate groups of aceticlastic, methylotrophic and hydrogenotrophic methanogens, the methylotrophs contain specific methyltransferases as has previously been reported,^53–55^ while hydrogenotrophs contain hydrogenases and dehydrogenases as described by Costa and Leigh.^56^ Interestingly, we find that aceticlastic methanogens have no unique genes related to methane metabolism (comprehensive list in Supplementary Table S3). This could indicate a general ability of methanogens to metabolize acetate, which has been observed in the hydrogenotroph *Methanococcus maripaludis*.^57^ In addition to considering variations with respect to carbon chemistry, Lyu and Lu^58^ showed genome content differences in Class I and Class II methanogens (those formerly known as Methanobacteriota and Halobacteriota), with the latter having greater oxidative adaptation abilities.

### Phylogenetic distance negatively correlates with shared orthogroups

**Figure 5:**
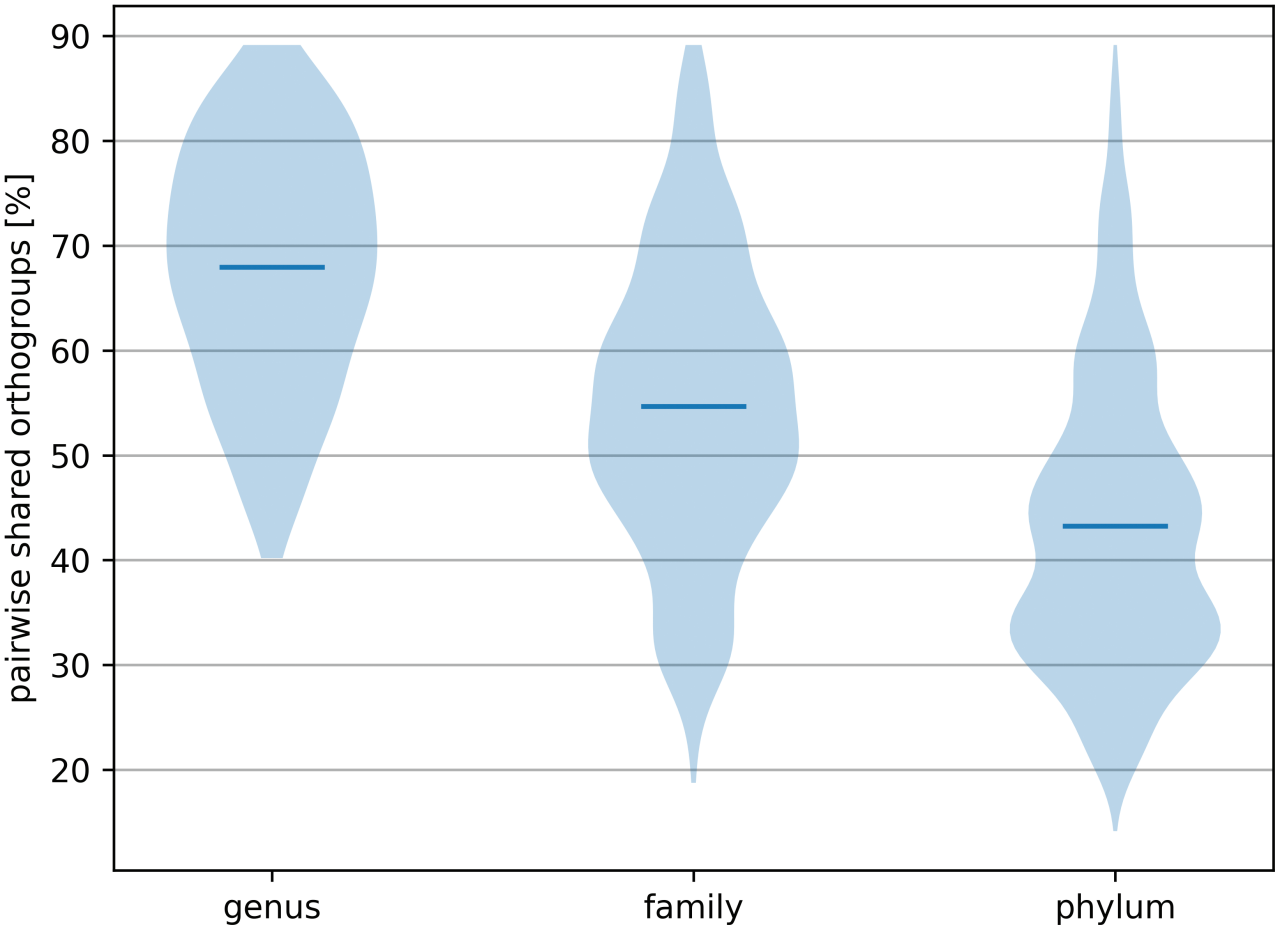
Pairwise shared orthogroups across clades. Species–species comparisons within each genus (*n* = 180 in 23 genera), family (*n* = 884 in 9 families) and phylum (*n* = 3640 in 2 phyla). The mean is marked with a solid line.

Across the phylogenetic diversity of methanogens, we compared the pairwise number of orthogroups shared between two genomes at the genus, family and phylum level. Excluding those clades with only one genome member, our comparisons include 180 pairwise comparisons in 23 genera, 884 in 12 families and 3640 in 2 phyla (Fig. 5). We find that within a genus, two species can share as little as 40% of their orthogroups (within the genus *Methanosarcina*) and a decrease in shared fractions with increasing phylogenetic distance. On average 67.95% of orthogroups are shared between two species within a genus, 54.66% of orthogroups are shared between two species in a family and 43.27% of orthogroups are shared between two species in a phylum. Our observations are related to those presented by Touchon et al.,^59^ who found that gene repertoire relatedness decreases with increasing phylogenetic distance, but more work is needed to understand how genome content varies at different taxonomic depths.

In a previous analysis including two hydrogenotrophic species from the *Methanothermobacter* genus,^23^ orthogroups shared between the two genomes were largely related to CO_2_ reduction and energy conservation, as well as the translocation of metals and ions other than sodium. Between those two species from the same genus, 85% and 91% of genomic content was shared respectively. In another study, Borton et al.^24^ showed that across eleven genomes within the genus *Methanohalophilus*, most core genes accounted for housekeeping and methylotrophic methanogenesis-related functions. The core accounted for only 42.5–49.4% of the total genome size, which may be attributed to the inclusion of MAGs that tend to have lower completeness and quality scores.

### Amino acid composition of the proteome

**Figure 6:**
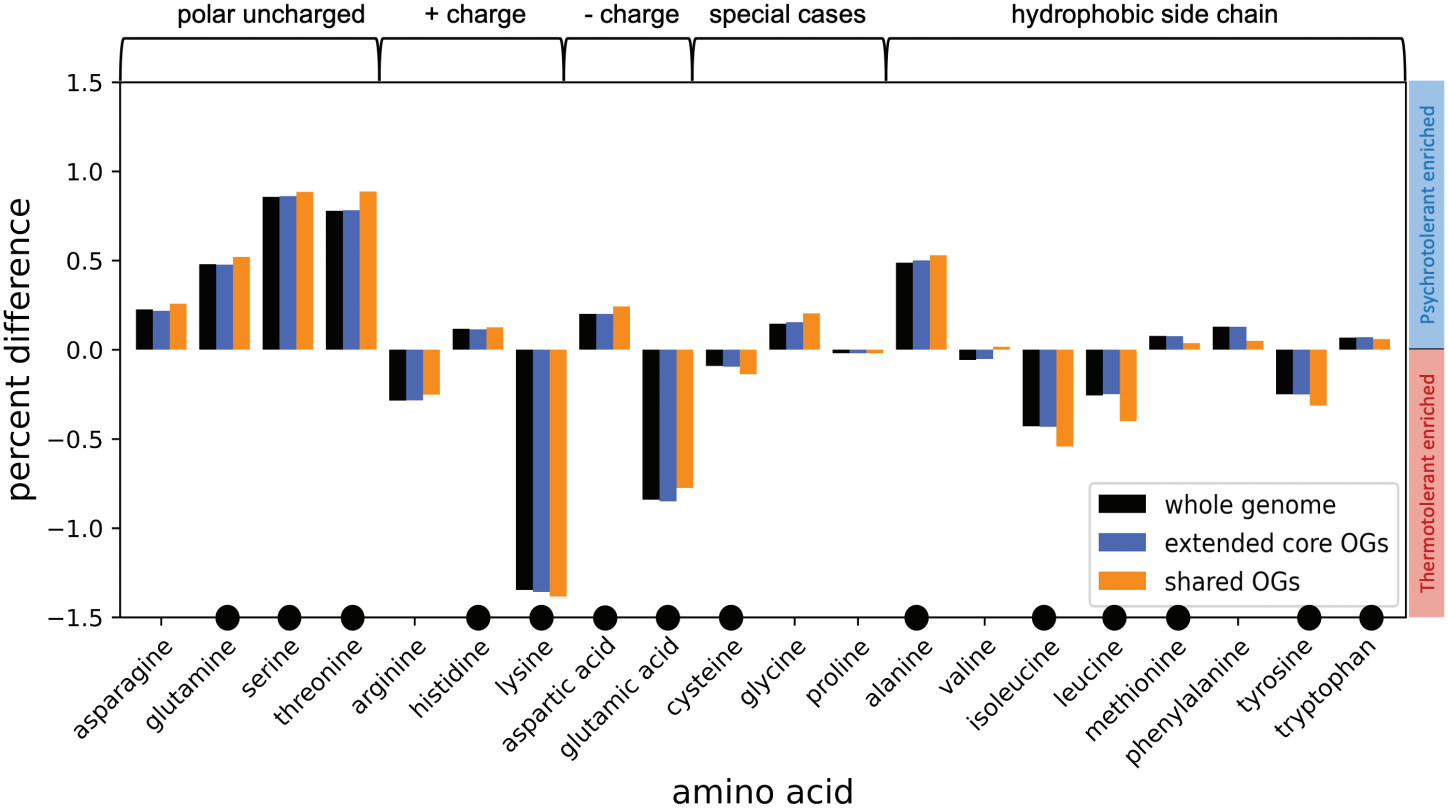
Amino acid compositional differences between temperature groups. Positive percentages indicate amino acid enrichment in psychrotolerant species, and negative percentages indicate amino acid enrichment in thermotolerant species. Percentages were obtained by averaging the relative amino acid abundance over the proteome/extended core orthogroups/shared orthogroups of the two temperature groups and subtracting the thermotolerant mean from the psychrotolerant mean. Circles denote amino acids that have significant correlation with temperature across the whole range of temperatures and for all 86 species, when optimal growth temperature was plotted against each amino acid’s abundance (Benjamini Hochberg correction on Pearson P-values). Amino acid abundances for each OG grouping can be found in Supplementary Table S4.

It has been suggested that compositional biases in amino acid content are not observable at the whole genome level,^60^ however for the methanogens, we observe compositional shifts when comparing methanogens at both the whole proteome and conserved extended core levels. Out of 20 amino acids, 14 show significant correlations with the optimum growth temperature of organisms, with slightly more pronounced differences in the shared proteome compared to the extended core (Fig. 6). Polar uncharged amino acids are increased in the psychrotolerant species, while lysine, glutamic acid, leucine and isoleucine are increased in thermotolerant species. In psychrotolerant organisms, amino acid substitutions increase protein flexibility and specific activity and decrease thermostability,^61,62^ while in thermotolerant organisms stability is increased.^63^

Our results are in line with Saunders et al.,^64^ who compared then available methanogen species (*n* = 9) and found an almost linear trend in the content of glutamine, threonine and leucine over the range of studied growth temperatures (15–98°C) with glutamine and threonine being more abundant and leucine being less abundant in cold-tolerant archaea. Our results are also in agreement with a previous study of the methanogenic genus *Methanococcus*, which found that amino acid biases are affected mostly by optimal growth temperature and not by phylogenetic distance,^65^ and most recently with the result of Lecocq et al.^22^ who found that amino acid frequency variation is the main driver in temperature adaptation in the order *Methanococcales.*

Interestingly, amino acid compositional trends were generally found to be uncorrelated between psychrotolerant and thermotolerant species analysed at the family level.^66^ Our results suggest that for the methanogens, a shared biochemical repertoire underlies coordinated amino acid changes across the growth temperature range.

**Figure 7.**
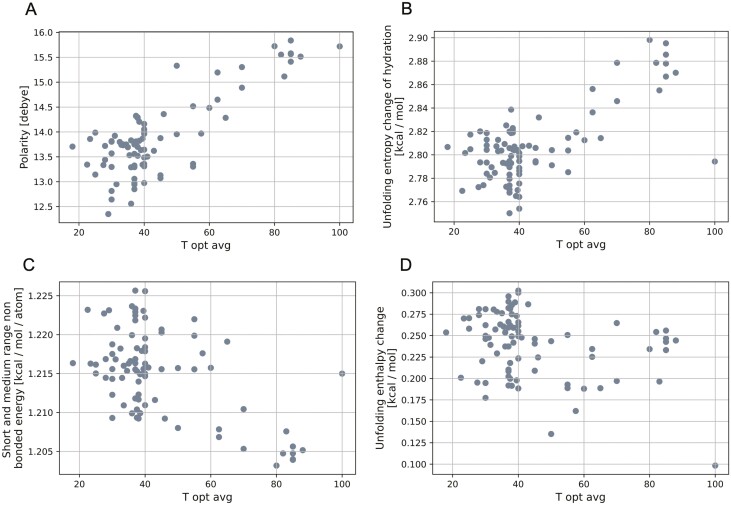
Selected proteome properties in relation to optimal growth temperature (°C): polarity (A), unfolding entropy change of hydration (B), short and medium range non bonded energy (C) and unfolding enthalpy change (D). Plots for other amino acid properties are available in Supplementary Fig. S6.

The amino acid composition of a proteome has a direct impact on its structural and energetic features: We find that the proportion of charged amino acids and hydrophobicity have positive correlations with optimum growth temperature, while amino acid properties associated with structural complexity such as turn and coil tendencies, have negative correlations with the optimum growth temperature (Fig. 7; Table 2). Comparing of unfolding entropy and enthalpy (Fig. 7B and C) reinforces our understanding that protein folding is entropically driven. Some of these properties have been described for thermophilic enzymes in Ando et al.,^67^ where the authors propose mechanisms that increase the stability of those enzymes, including entropic stabilization.^68^ Interestingly, although the average isoelectric point of amino acids in the proteome increases with increasing temperature, the charge state of the amino acids across the whole proteomes of hyperthermophilic methanogens may be closer to zero compared to their mesophilic relatives since the average amino acid isoelectric point is closer to neutral water at higher temperatures (Supplementary Fig. S6; recall that neutral pH changes from 7 at 25°C to 6.1 at 100°C^1^). It is also possible that the increase in lysine observed in thermotolerant organisms drives the increase in proteome isoelectric point (Fig. 7).

**Table 2.** Proteome amino acid properties with temperature correlations

Increase with temperature	Decrease with temperature
Isoelectric point (0.812)	Coil tendencies (−0.677)
Polarity (0.808)	Turn tendencies (−0.617)
Refractive index (0.737)	Short and medium range non bonded energy (−0.567)
Thermodynamic transfer hydrophobicity (0.715)	Sombined surrounding hydrophobicity globular and membrane (−0.356)
Unfolding entropy change of hydration (0.690)	Total non-bonded energy (−0.350)
Unfolding hydration heat capacity change (0.686)	Unfolding enthalpy change (−0.318)
Helical contact area (0.675)	Unfolding enthalpy change of hydration (−0.311)
Average medium contacts (0.637)	Buriedness (−0.264)
Solvent accessible surface area for denatured protein (0.622)	Compressibility (−0.251)
Flexibility (0.579)	Chromatographic index (−0.225)
Solvent accessible reduction ratio (0.561)	Unfolding Gibbs free energy change (−0.218)
Bulkiness (0.560)	Long range non-bonded energy (–0.058)
Volume (0.535)	
Molecular weight (0.528)	
Solvent accessible surface area for native protein (0.523)	
Unfolding entropy change (0.339)	
Shape (0.241)	

Protein amino acid properties with Significant correlation to temperature. The Pearson correlation coefficient for each property is in parentheses. Scatterplots showing the data are available in Supplementary Fig. S6.

An important and interesting outlier in our study is *Methanopyrus kandleri,* which grows at the highest temperature of the methanogens at 122°C. Surprisingly, this organism exhibits many proteome properties, such as bulkiness, flexibility, helical contact area, solvent accessible surface area and volume, more similar to mesophilic than other thermotolerant organisms (Supplementary Fig. S6; *Methanopyrus kandleri* is found near the 100°C mark of its optimal temperature). This observation raises questions about the mechanisms of thermal adaptation and the use of amino acid profiles to predict growth temperature. For example, some studies propose the use of certain amino acid ratios (e.g. charged vs. polar amino acids) to predict optimal growth temperatures of un-cultured organisms,^69^ but *Methanopyrus kandleri* seems to challenge our ability to make generalizations. Although it is unknown how *Methanopyrus kandleri* can live at high temperatures without altering its proteome amino acid composition as other methanogens do, it is tempting to speculate that the organism may have an altered cytosol content in comparison to other methanogens, but work is needed to clarify this. We note that protein compositional differences are associated with other environmental variables other than temperature; for example, pH, oxidation state^70^ and salinity.^71^ Future work is needed to build an integrative picture of how multiple variables affect the amino acid composition of the proteome.

### Gene gain and loss in methanogens

We used a species tree of the 86 species to investigate a possible phylogenetic relationship between gene gain–loss-duplication with temperature. Although the thermotolerant organisms have smaller genomes, correlations between temperature and gain-loss-duplication were not observed (Supplementary Fig. S7). This observation leaves open questions as to how the temperature:genome size relationship (Fig. 4A) came to be.

Thomas et al.^72^ showed that in comparison with 21 other Methanosarcinales, the genome of the rumen dwelling and mesophilic methanogen *Methanosarcina blatticola* shows the largest genome reduction, with the loss of almost all genes involved in the H4MPT (tetrahydromethanopterin) methyl branch of the Wood–Ljungdahl pathway. In line with this, our analysis shows that *Methanosarcina blatticola* has the highest reduction with a net gain rate of −0.36, while other Methanosarcinales show both net gains and losses. This clade-specific variance within the Methanosarcinales suggests that gene gain–loss-duplication rates are not solely governed by the phylogenetic placement of organisms, and it may be that this type of variability is part of what clouds our ability to observe a temperature gain-loss-duplication rate relationship.

## Conclusions

We observed differences in genome size and functional composition across the genomes of methanogens spanning a wide growth temperature range. We show that differences in amino acid composition can be found at the whole genome level as well as in the functionally constant extended core. Thermotolerant methanogens have smaller, reduced genomes with an increase in charged amino acids and functional genes related to ion transport which are not found in their psychrotolerant counterparts. The latter have larger genomes with an increase in polar uncharged amino acids and additional functional genes related to cell structure and mobility. The organisms in our core genome analysis span three phyla but share a large extended core, suggesting that physiological similarity has a stronger effect on temperature adaptation than phylogenetic distance in the methanogens. We show that core size decreases with the increased phylogenetic distance between the methanogenic archaea, and it will be interesting to explore this trend across the tree of life.

The expansion of our knowledge of alkane-utilizing archaea, their phylogenies, metabolisms and genomic features has widened our understanding of archaeal evolutionary history in the past decade.^7,69,73–80^ Further studies focusing on their genome evolution can help us further narrow down the first appearances of methanogens and methanotrophs in Earth’s history. The type of study conducted here could be expanded across a greater diversity of organisms, further helping to clarify how environment, physiology and evolution each contribute to genomic content.

## Supplementary Material

dsac048_suppl_Supplementary_Figures

dsac048_suppl_Supplementary_Tables
