# Supplementary material for: The methanogen core and pangenome: conservation and variability across biology’s growth temperature extremes
Source: DNA Res. 2022 Dec 1;30(1):dsac048. doi: 10.1093/dnares/dsac048 (PMC9886072; doi:10.1093/dnares/dsac048)

Supplementary Figures for: The methanogen core and pangenome: conservation and variability across biology's growth temperature extremes

***Paula Prondzinsky, Sakae Toyoda, *Shawn Erin McGlynn**

*corresponding authors: prondzinsky@elsi.jp; mcglynn@elsi.jp

Figure S1: Temperature ranges for each of the 86 organisms used in the main analysis. Vertical lines indicate the cutoff temperatures for psychrotolerant (15 °C) and thermotolerant (45 °C) groupings. Organisms spanning across both lines are considered to be mesophilic.

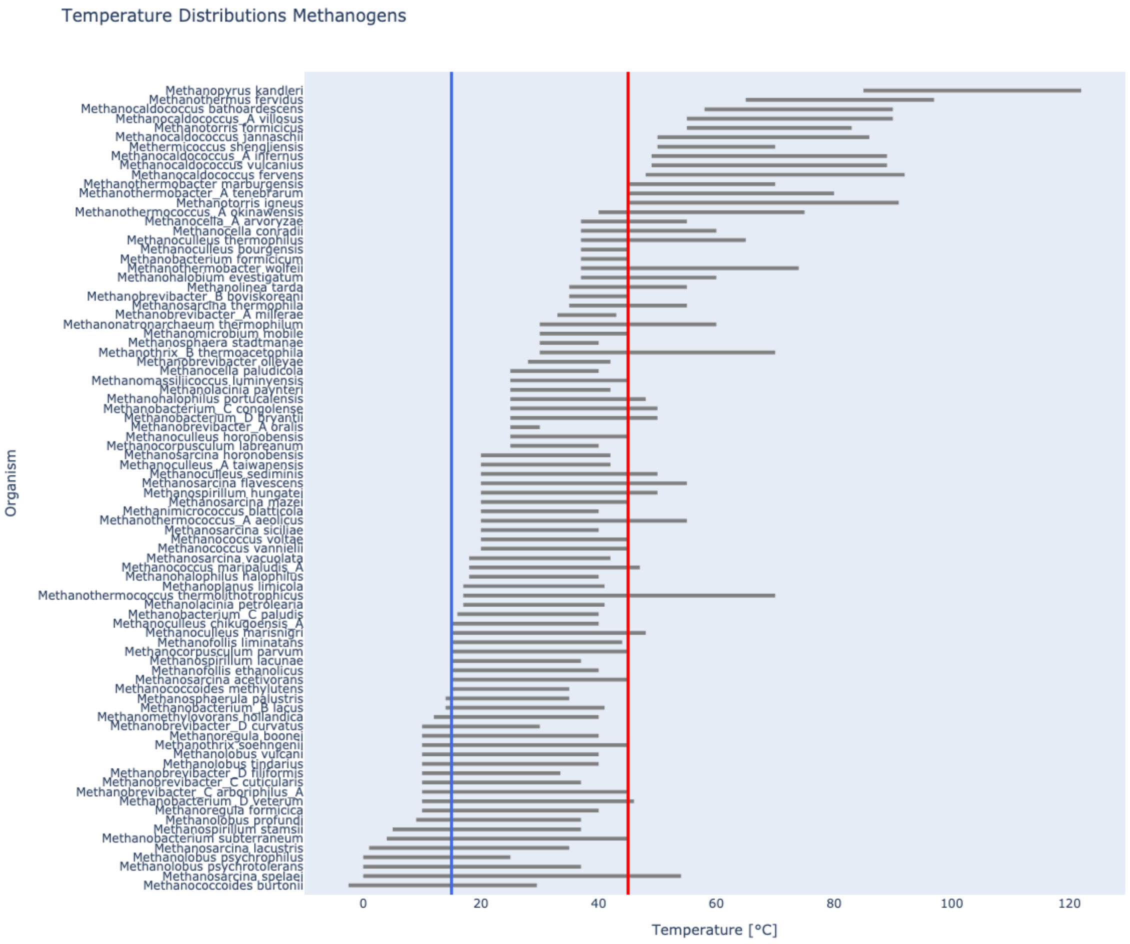

Figure S2: Branch lengths from the root of the archaeal tree to each species with their respective minimum (A) and maximum (B) growth temperatures.

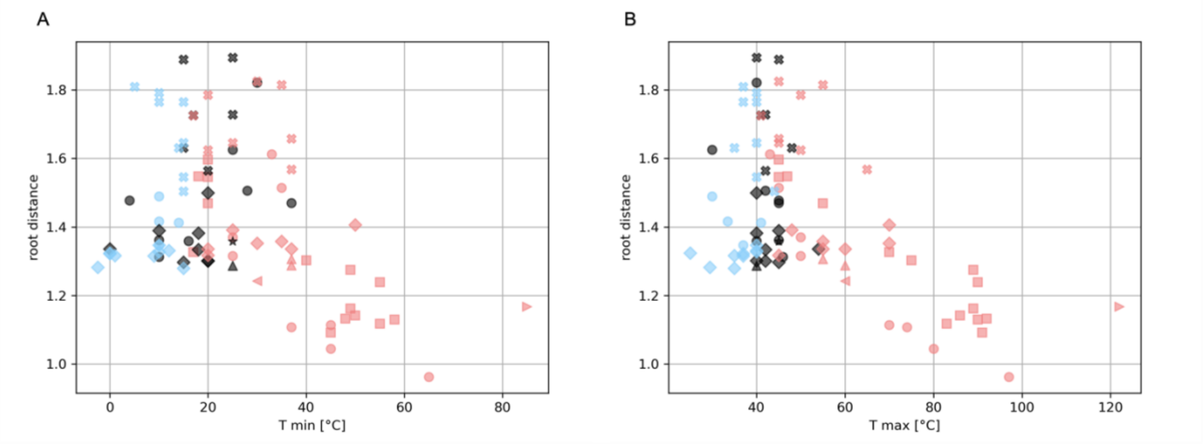

Figure S3: Distributions of genome sizes with minimum (A) and maximum (B) growth temperatures.

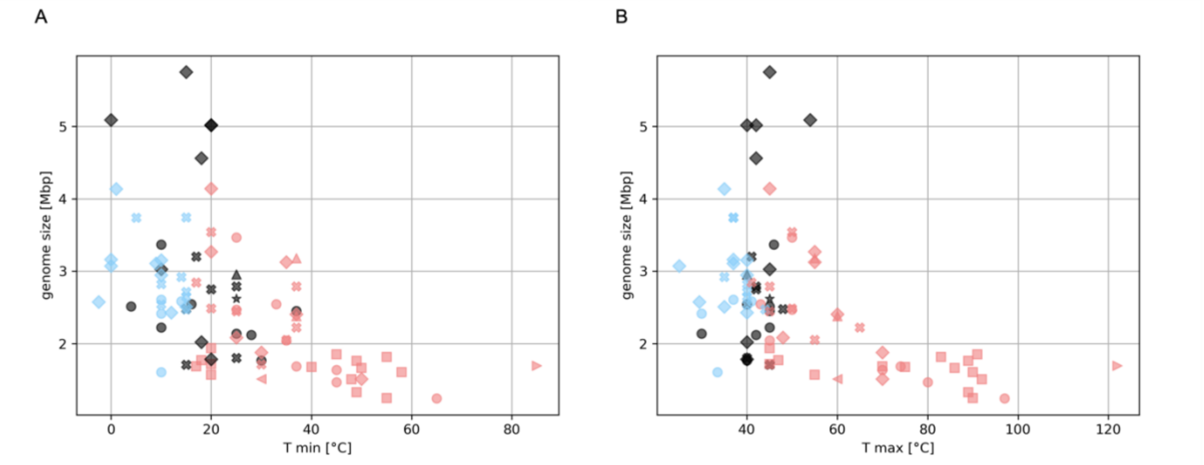

Figure S4: species tree for 86 methanogenic species with temperature data. Colored rings indicate minimum and maximum observed growth temperatures, shapes indicated methanogenesis pathways. Red shaded branches are species in the thermotolerant group, blue shaded branches are species in the psychrotolerant group. Uncolored branches are in neither temperature group and considered as mesophilic.

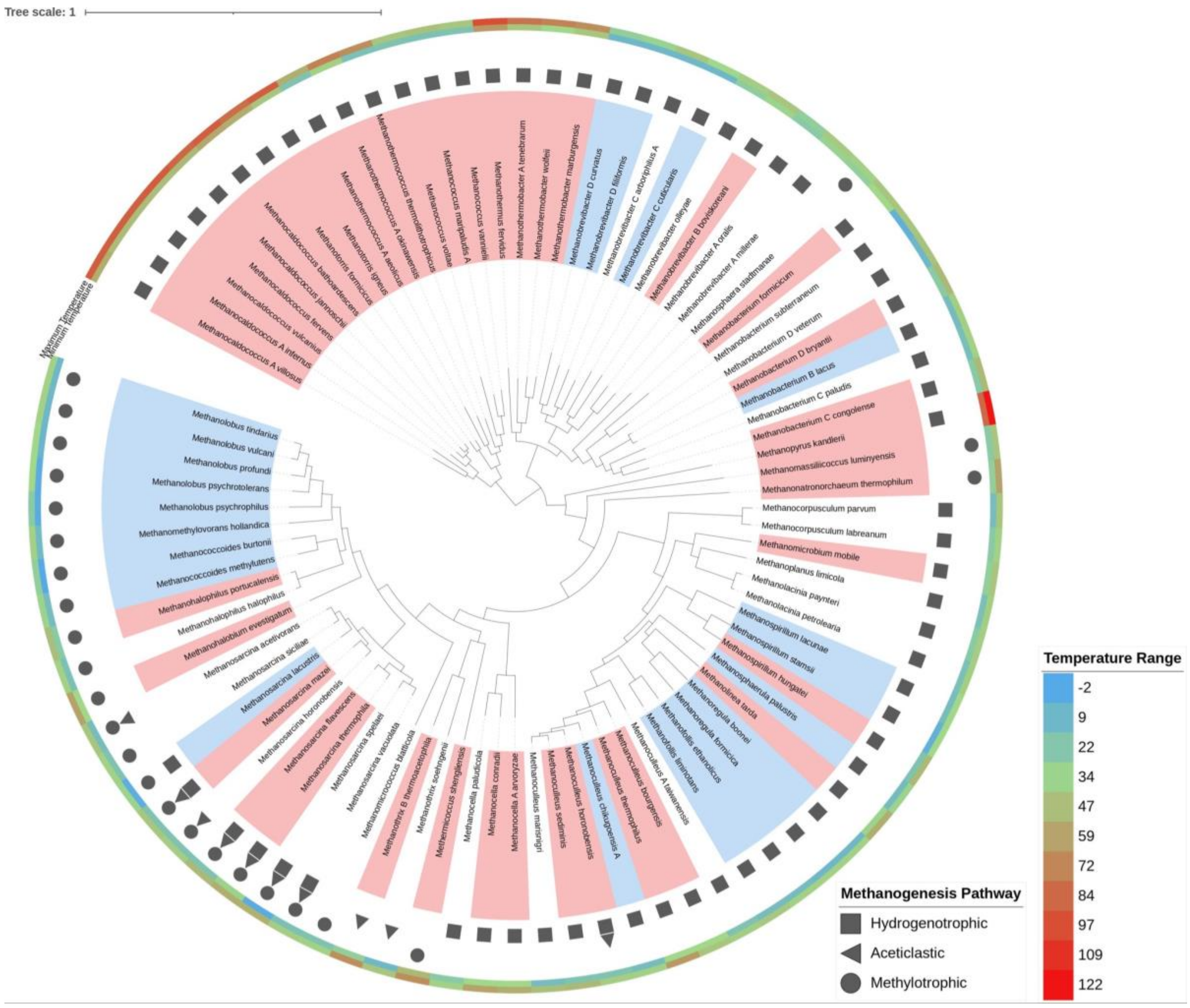

Figure S5: 86 species COG functional categories for (A) the extended core, (B) shared orthogroups and (C) unique orthogroups.

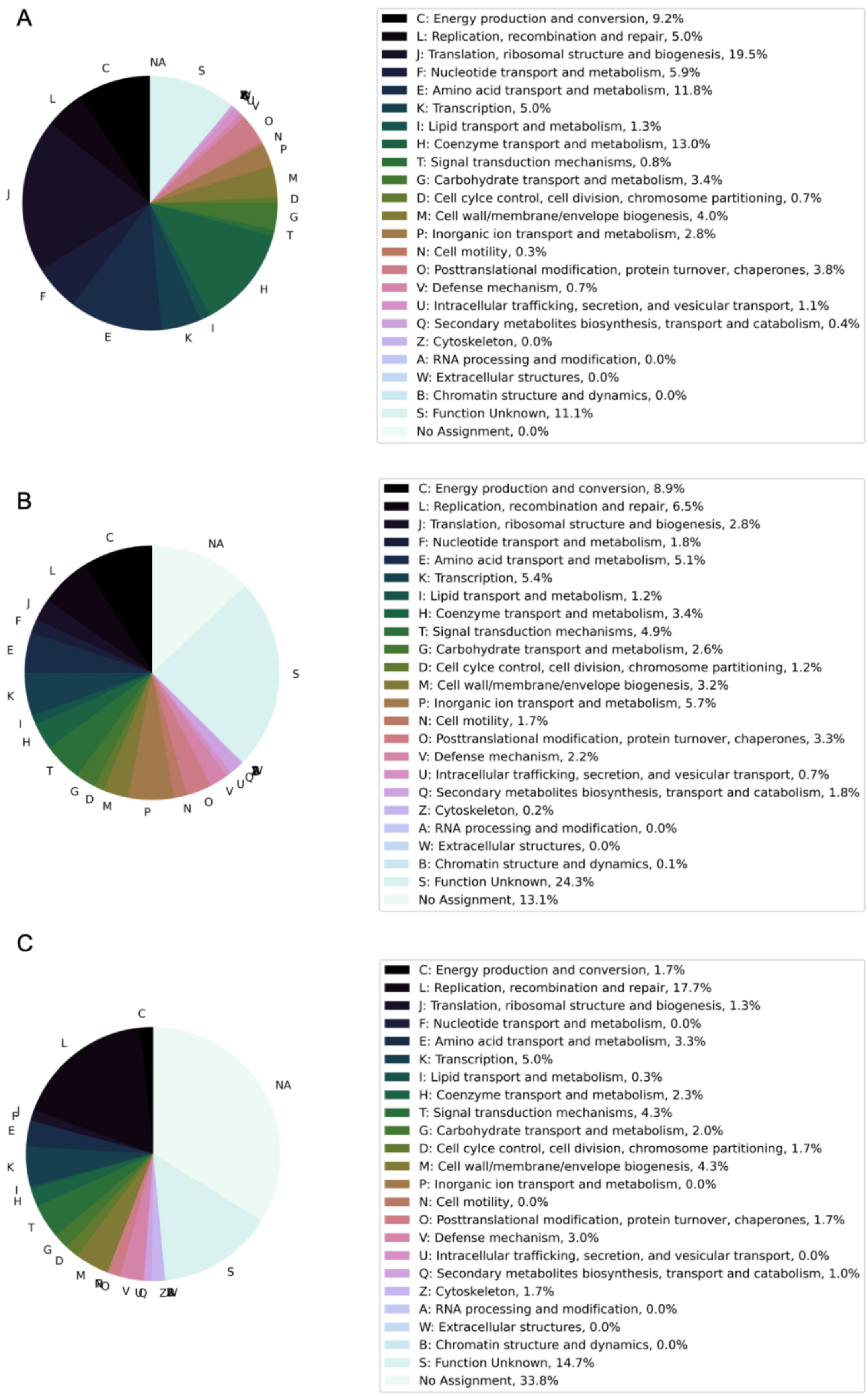
A
C: Energy production and conversion, 9.2%
L: Replication, recombination and repair, 5.0%
J: Translation, ribosomal structure and biogenesis, 19.5%
F: Nucleotide transport and metabolism, 5.9%
E: Amino acid transport and metabolism, 11.8%
K: Transcription, 5.0%
I: Lipid transport and metabolism, 1.3%
H: Coenzyme transport and metabolism, 13.0%
T: Signal transduction mechanisms, 0.8%
G: Carbohydrate transport and metabolism, 3.4%
D: Cell cylce control, cell division, chromosome partitioning, 0.7%
M: Cell wall/membrane/envelope biogenesis, 4.0%
P: Inorganic ion transport and metabolism, 2.8%
N: Cell motility, 0.3%
O: Posttranslational modification, protein turnover, chaperones, 3.8%
V: Defense mechanism, 0.7%
U: Intracellular trafficking, secretion, and vesicular transport, 1.1%
Q: Secondary metabolites biosynthesis, transport and catabolism, 0.4%
Z: Cytoskeleton, 0.0%
A: RNA processing and modification, 0.0%
W: Extracellular structures, 0.0%
B: Chromatin structure and dynamics, 0.0%
S: Function Unknown, 11.1%
No Assignment, 0.0%
B
C: Energy production and conversion, 8.9%
L: Replication, recombination and repair, 6.5%
J: Translation, ribosomal structure and biogenesis, 2.8%
F: Nucleotide transport and metabolism, 1.8%
E: Amino acid transport and metabolism, 5.1%
K: Transcription, 5.4%
I: Lipid transport and metabolism, 1.2%
H: Coenzyme transport and metabolism, 3.4%
T: Signal transduction mechanisms, 4.9%
G: Carbohydrate transport and metabolism, 2.6%
D: Cell cylce control, cell division, chromosome partitioning, 1.2%
M: Cell wall/membrane/envelope biogenesis, 3.2%
P: Inorganic ion transport and metabolism, 5.7%
N: Cell motility, 1.7%
O: Posttranslational modification, protein turnover, chaperones, 3.3%
V: Defense mechanism, 2.2%
U: Intracellular trafficking, secretion, and vesicular transport, 0.7%
Q: Secondary metabolites biosynthesis, transport and catabolism, 1.8%
Z: Cytoskeleton, 0.2%
A: RNA processing and modification, 0.0%
W: Extracellular structures, 0.0%
B: Chromatin structure and dynamics, 0.1%
S: Function Unknown, 24.3%
No Assignment, 13.1%
C
C: Energy production and conversion, 1.7%
L: Replication, recombination and repair, 17.7%
J: Translation, ribosomal structure and biogenesis, 1.3%
F: Nucleotide transport and metabolism, 0.0%
E: Amino acid transport and metabolism, 3.3%
K: Transcription, 5.0%
I: Lipid transport and metabolism, 0.3%
H: Coenzyme transport and metabolism, 2.3%
T: Signal transduction mechanisms, 4.3%
G: Carbohydrate transport and metabolism, 2.0%
D: Cell cylce control, cell division, chromosome partitioning, 1.7%
M: Cell wall/membrane/envelope biogenesis, 4.3%
P: Inorganic ion transport and metabolism, 0.0%
N: Cell motility, 0.0%
O: Posttranslational modification, protein turnover, chaperones, 1.7%
V: Defense mechanism, 3.0%
U: Intracellular trafficking, secretion, and vesicular transport, 0.0%
Q: Secondary metabolites biosynthesis, transport and catabolism, 1.0%
Z: Cytoskeleton, 1.7%
A: RNA processing and modification, 0.0%
W: Extracellular structures, 0.0%
B: Chromatin structure and dynamics, 0.0%
S: Function Unknown, 14.7%
No Assignment, 33.8%

Supplementary Figure S6: Amino acid properties with $T_{opt\ avg}$; units as indicated in axes, otherwise dimensionless quantities

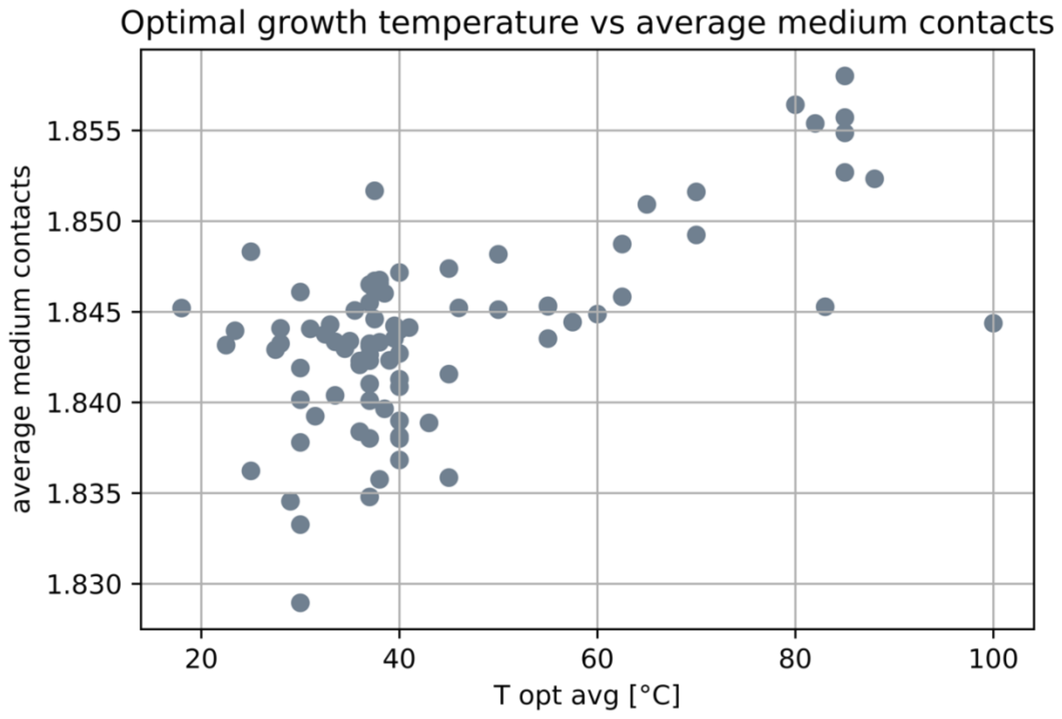

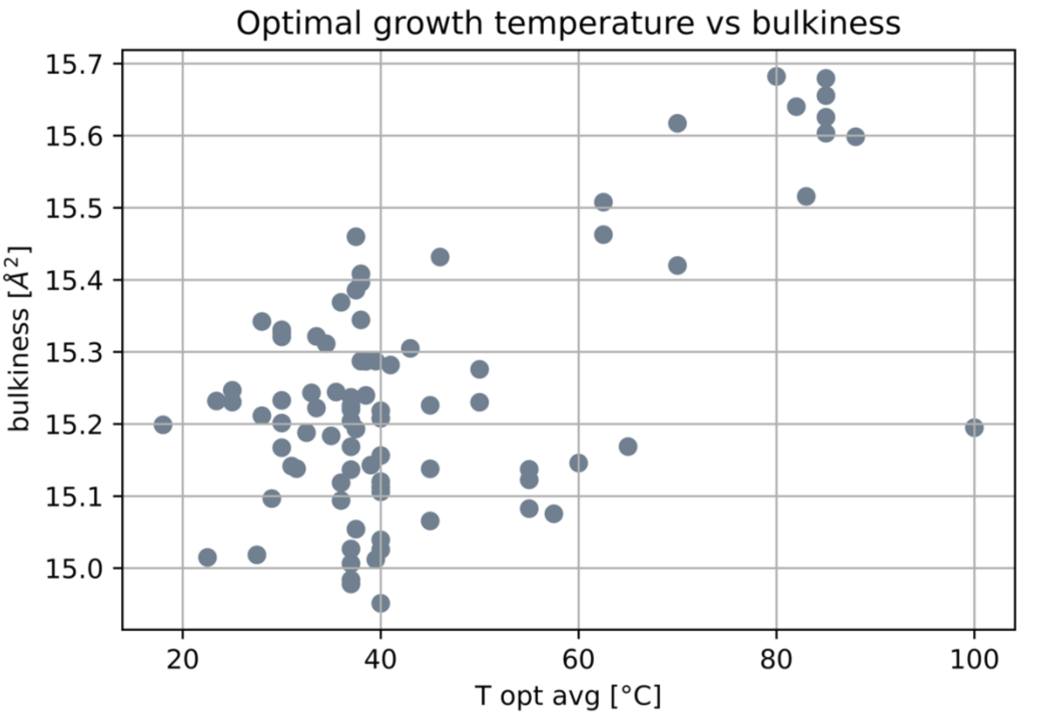

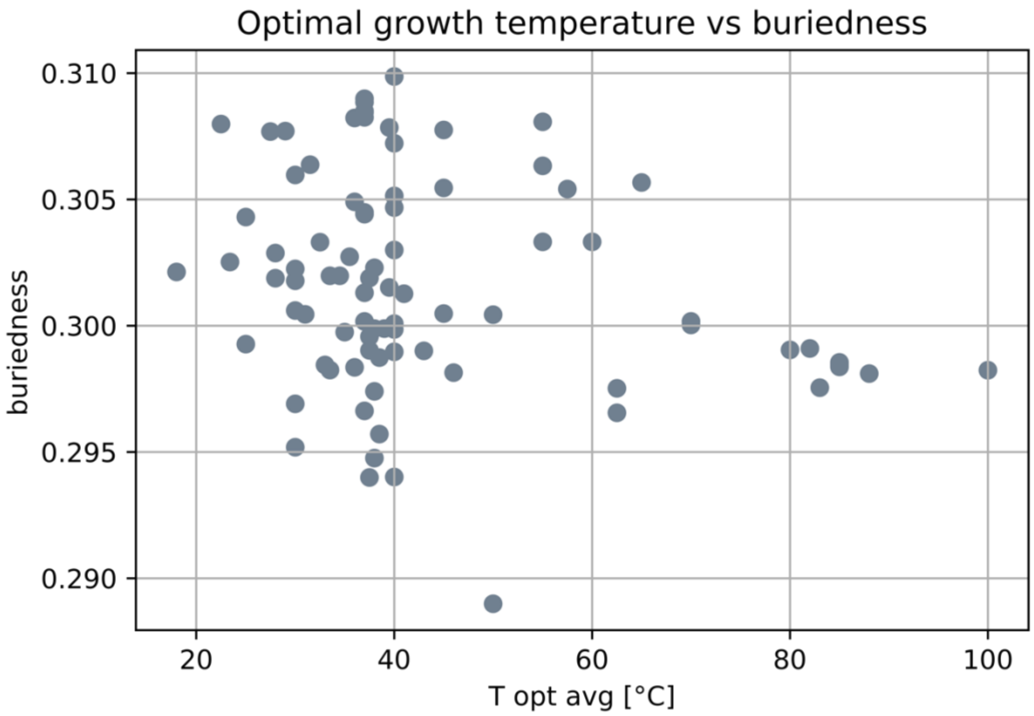
Optimal growth temperature vs buriedness
buriedness
0.310
0.305
0.300
0.295
0.290
20
40
60
80
100
T opt avg [°C]

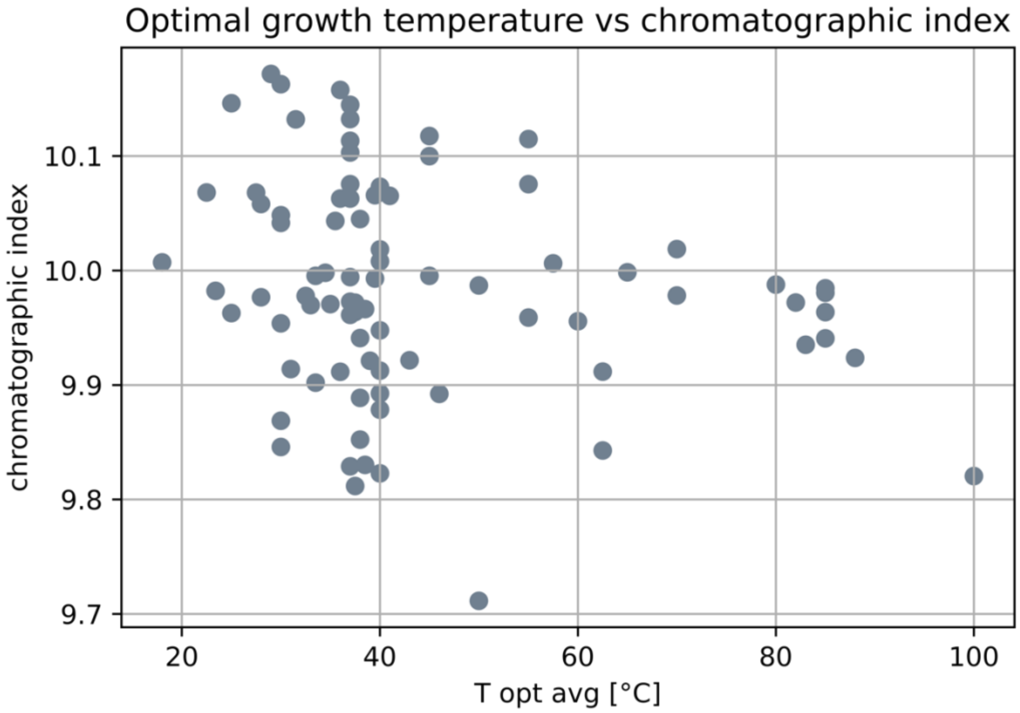
Optimal growth temperature vs chromatographic index
chromatographic index
10.1
10.0
9.9
9.8
9.7
20
40
60
80
100
T opt avg [°C]

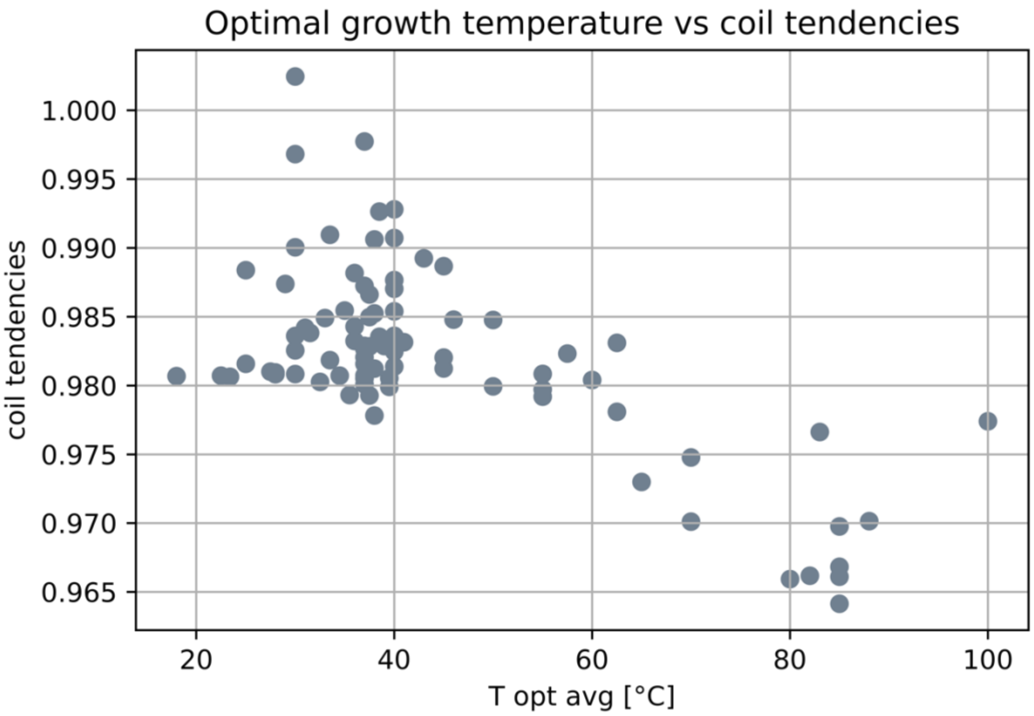
Optimal growth temperature vs coil tendencies
coil tendencies
1.000
0.995
0.990
0.985
0.980
0.975
0.970
0.965
20
40
60
80
100
T opt avg [°C]

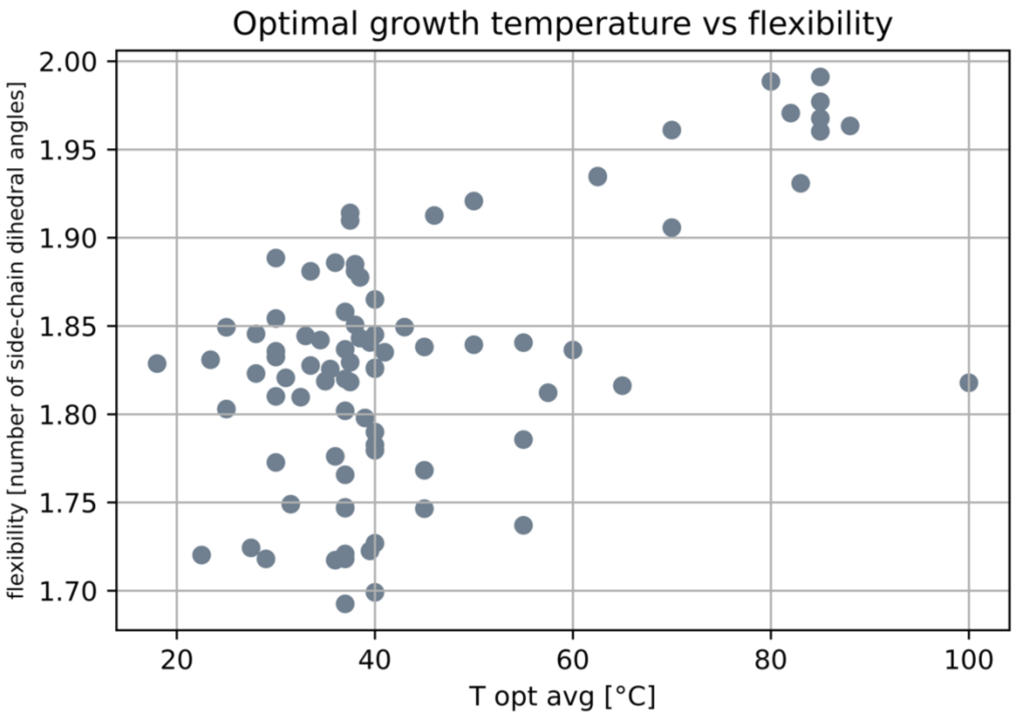
Optimal growth temperature vs flexibility
flexibility [number of side-chain dihedral angles]
2.00
1.95
1.90
1.85
1.80
1.75
1.70
20
40
60
80
100
T opt avg [°C]

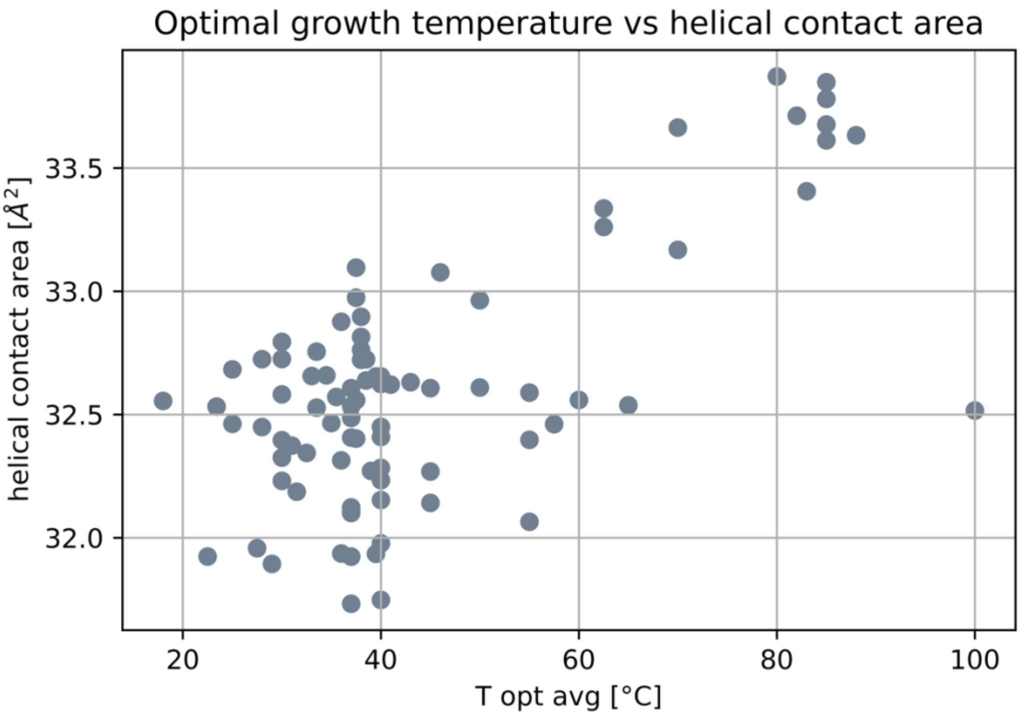

Optimal growth temperature vs helical contact area
helical contact area [Å^2]
33.5
33.0
32.5
32.0
20
40
60
80
100
T opt avg [°C]

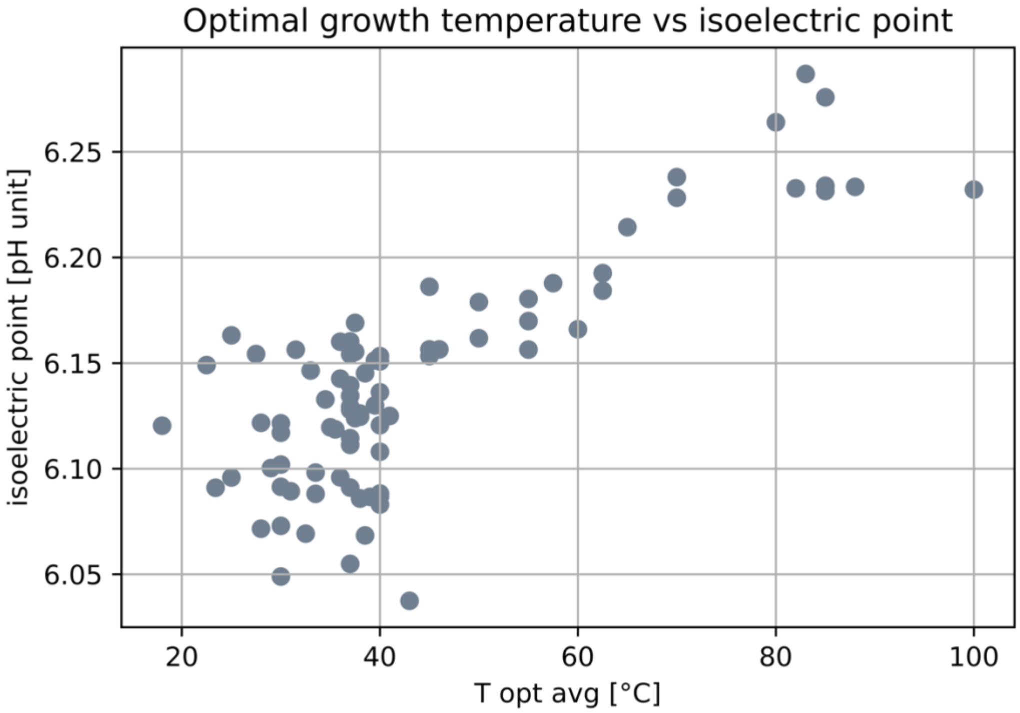

Optimal growth temperature vs isoelectric point
isoelectric point [pH unit]
6.25
6.20
6.15
6.10
6.05
20
40
60
80
100
T opt avg [°C]

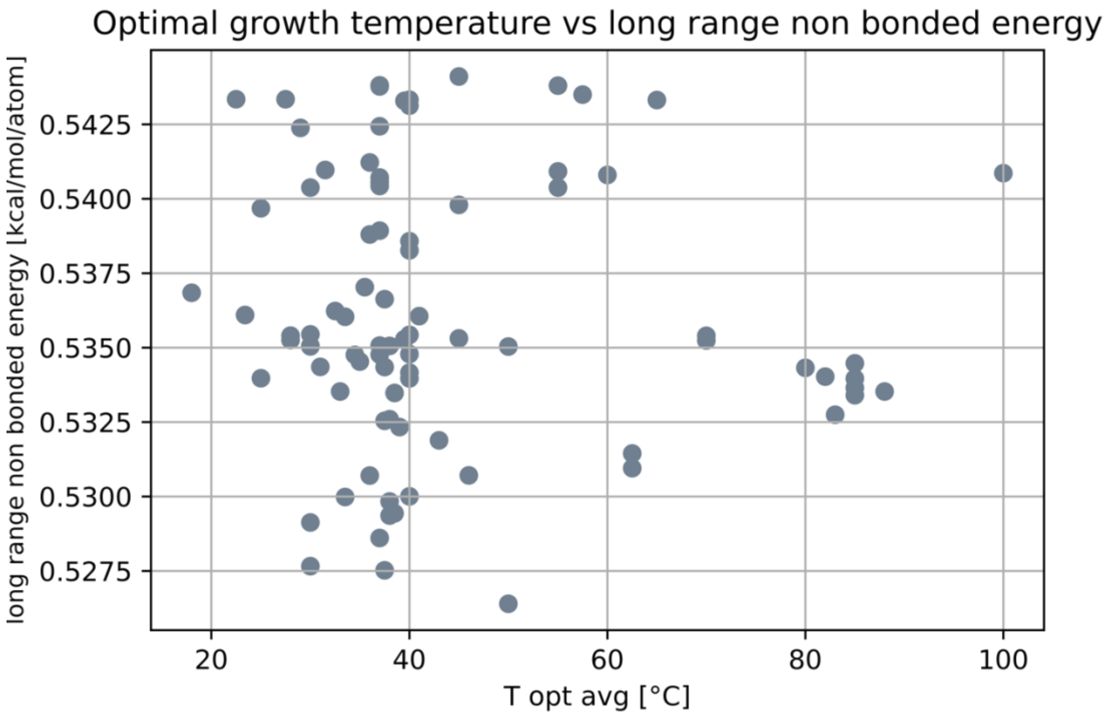
Optimal growth temperature vs long range non bonded energy
long range non bonded energy [kcal/mol/atom]
0.5425
0.5400
0.5375
0.5350
0.5325
0.5300
0.5275
20
40
60
80
100
T opt avg [°C]

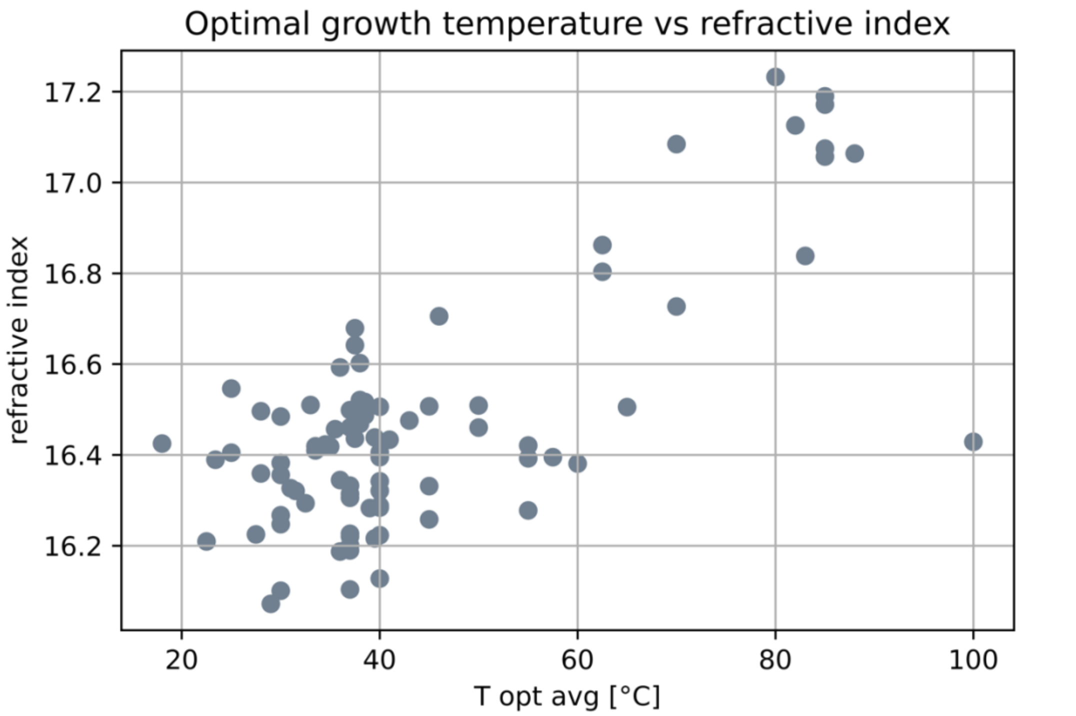
Optimal growth temperature vs refractive index
refractive index
17.2
17.0
16.8
16.6
16.4
16.2
20
40
60
80
100
T opt avg [°C]

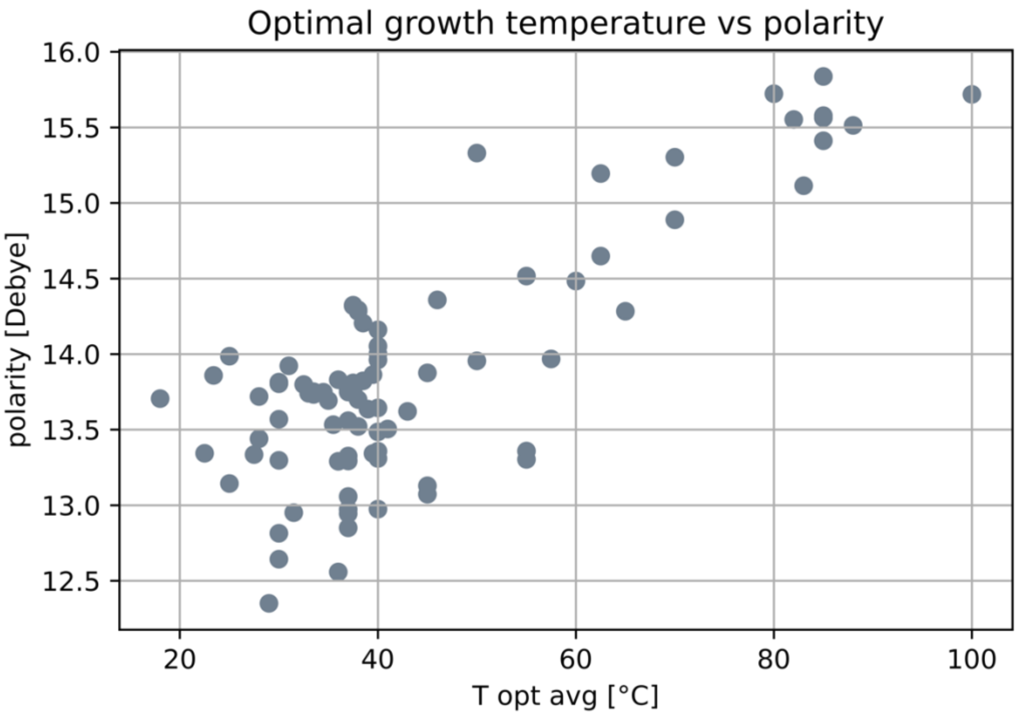
Optimal growth temperature vs polarity
polarity [Debye]
T opt avg [°C]
16.0
15.5
15.0
14.5
14.0
13.5
13.0
12.5
20
40
60
80
100

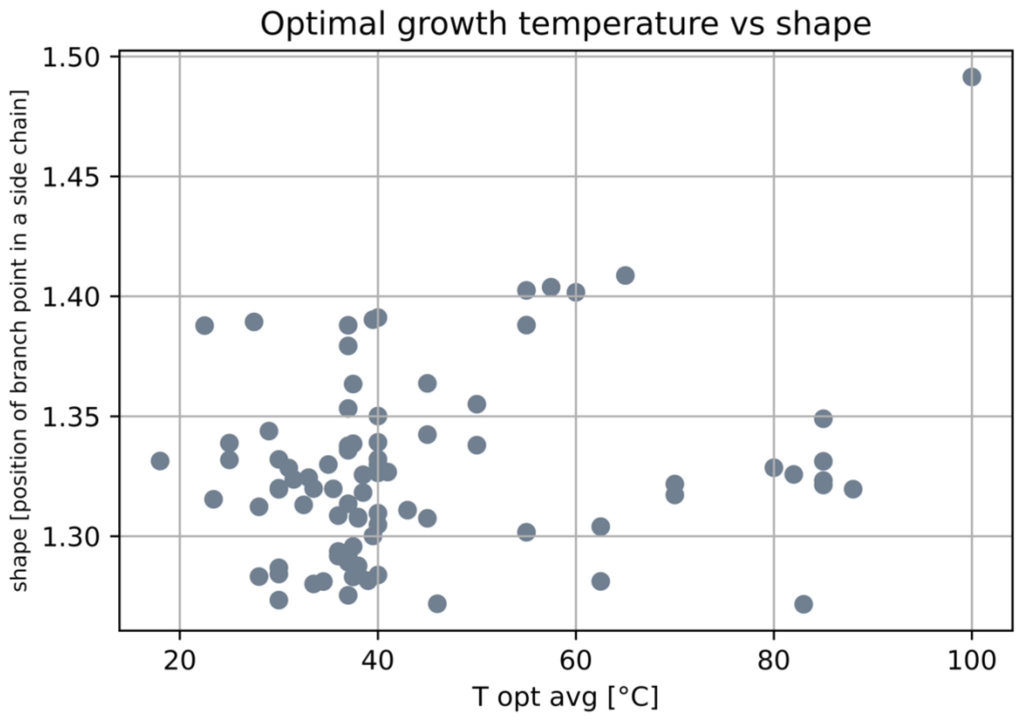
Optimal growth temperature vs shape
shape [position of branch point in a side chain]
T opt avg [°C]
1.50
1.45
1.40
1.35
1.30
20
40
60
80
100

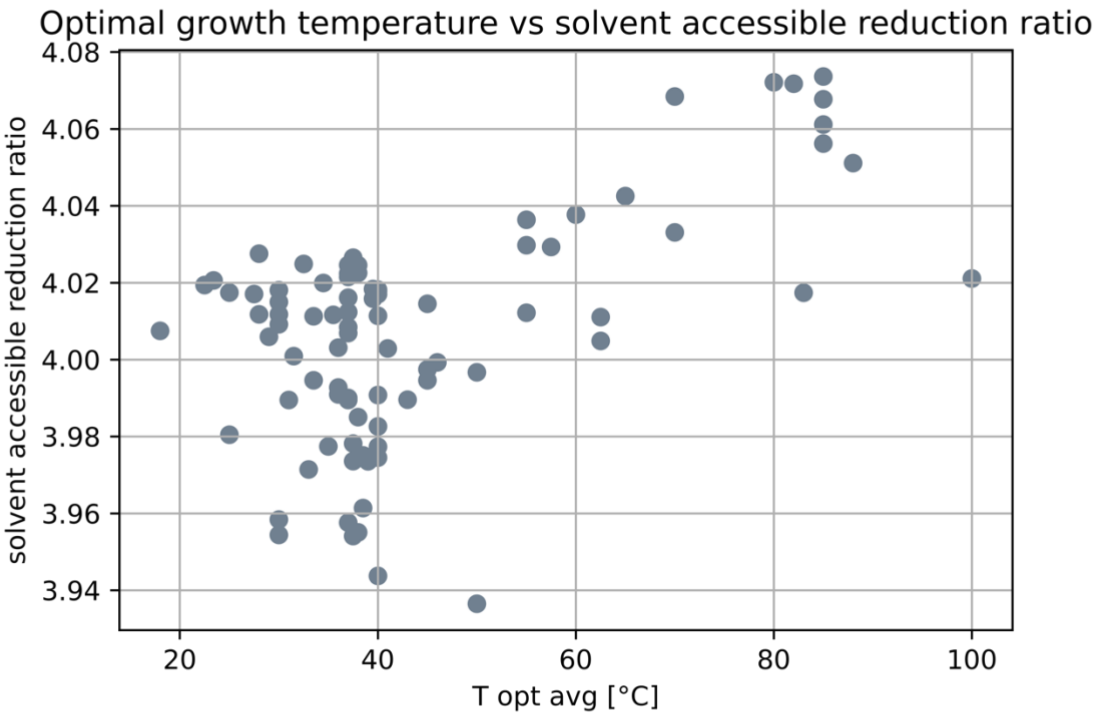

Optimal growth temperature vs solvent accessible reduction ratio
solvent accessible reduction ratio
4.08
4.06
4.04
4.02
4.00
3.98
3.96
3.94
20
40
60
80
100
T opt avg [°C]

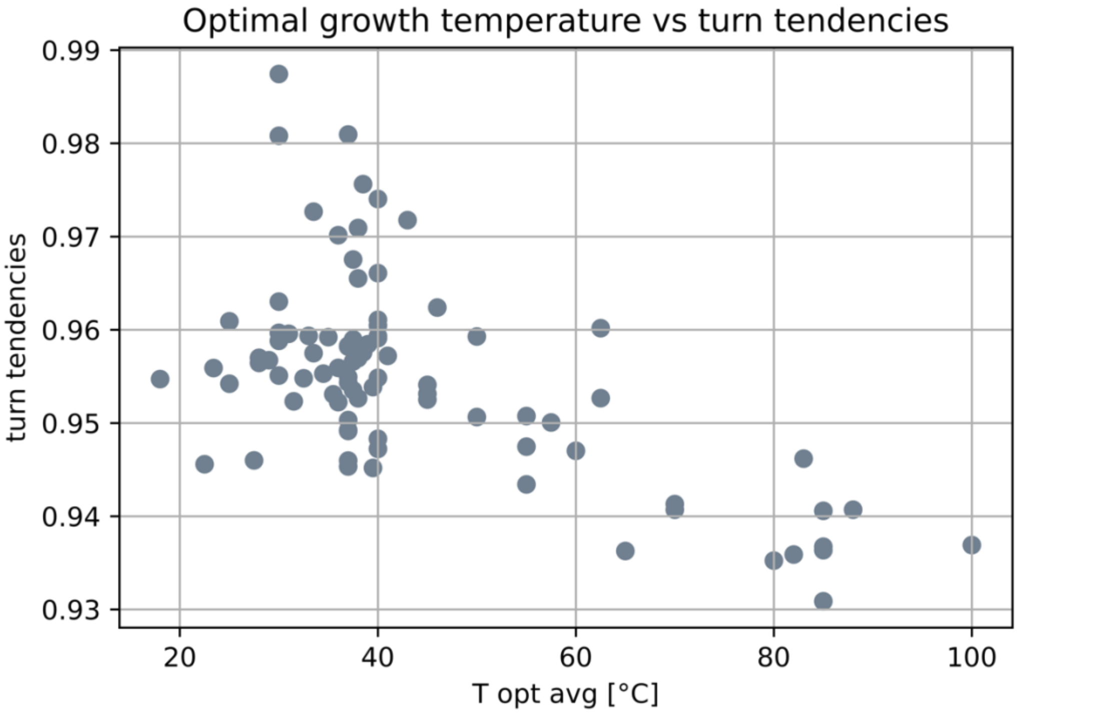

Optimal growth temperature vs turn tendencies
turn tendencies
0.99
0.98
0.97
0.96
0.95
0.94
0.93
20
40
60
80
100
T opt avg [°C]

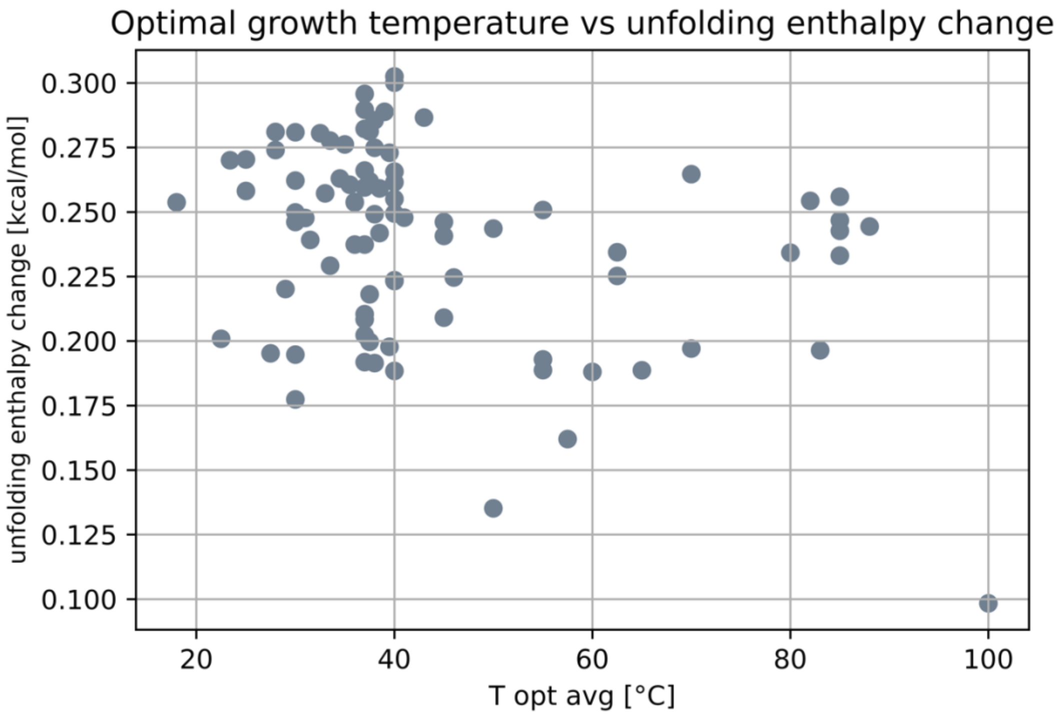
Optimal growth temperature vs unfolding enthalpy change
unfolding enthalpy change [kcal/mol]
0.300
0.275
0.250
0.225
0.200
0.175
0.150
0.125
0.100
20
40
60
80
100
T opt avg [°C]

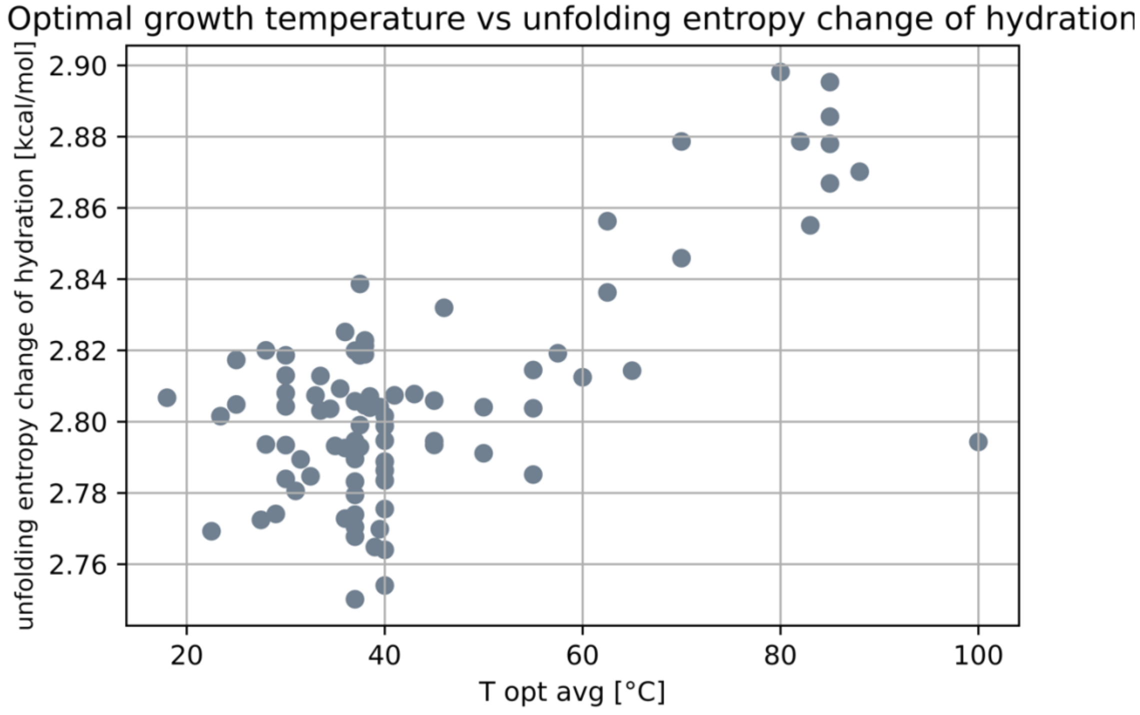
Optimal growth temperature vs unfolding entropy change of hydration
unfolding entropy change of hydration [kcal/mol]
2.90
2.88
2.86
2.84
2.82
2.80
2.78
2.76
20
40
60
80
100
T opt avg [°C]

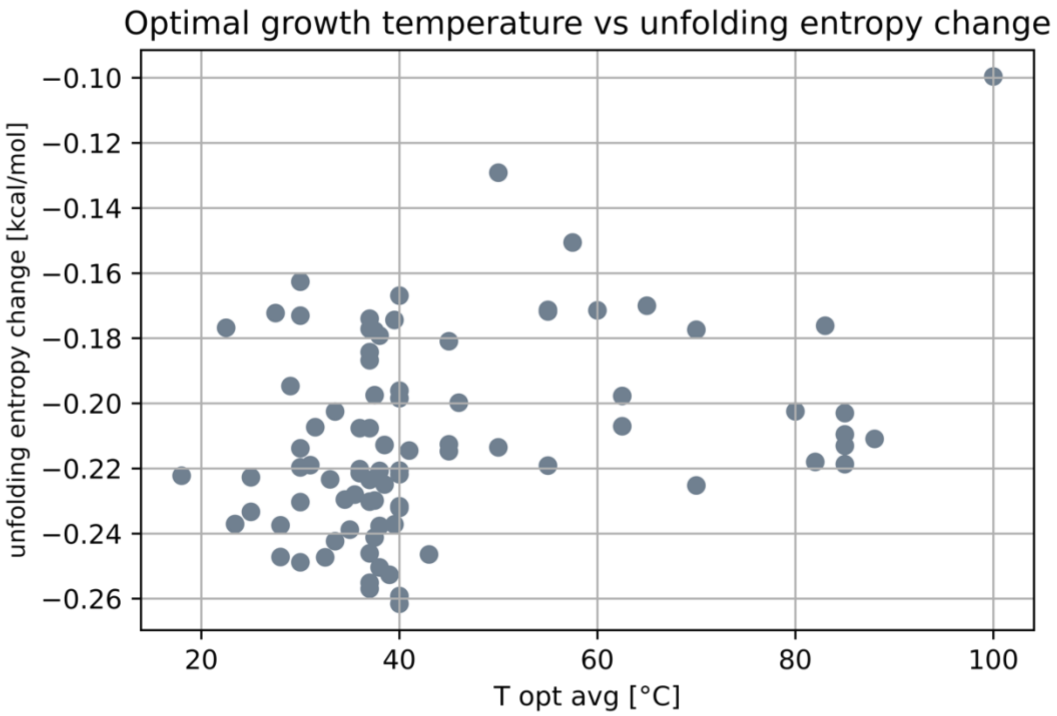

Optimal growth temperature vs unfolding entropy change
unfolding entropy change [kcal/mol]
−0.10
−0.12
−0.14
−0.16
−0.18
−0.20
−0.22
−0.24
−0.26
20
40
60
80
100
T opt avg [°C]

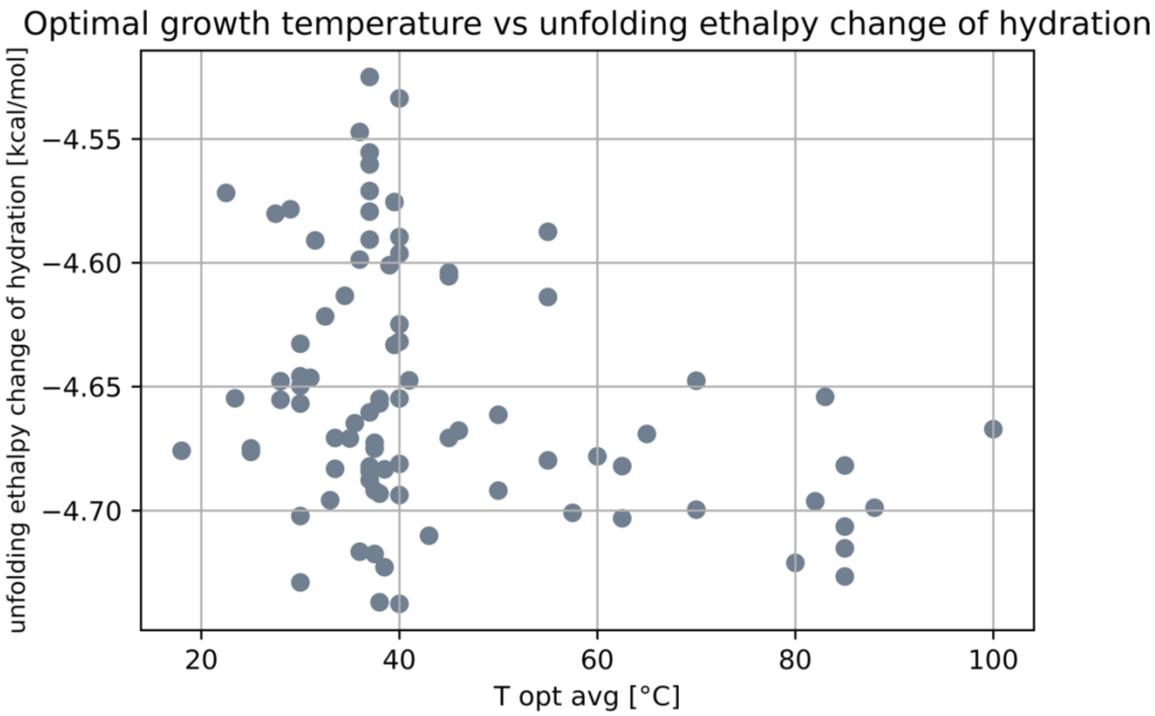

Optimal growth temperature vs unfolding ethalpy change of hydration
unfolding ethalpy change of hydration [kcal/mol]
−4.55
−4.60
−4.65
−4.70
20
40
60
80
100
T opt avg [°C]

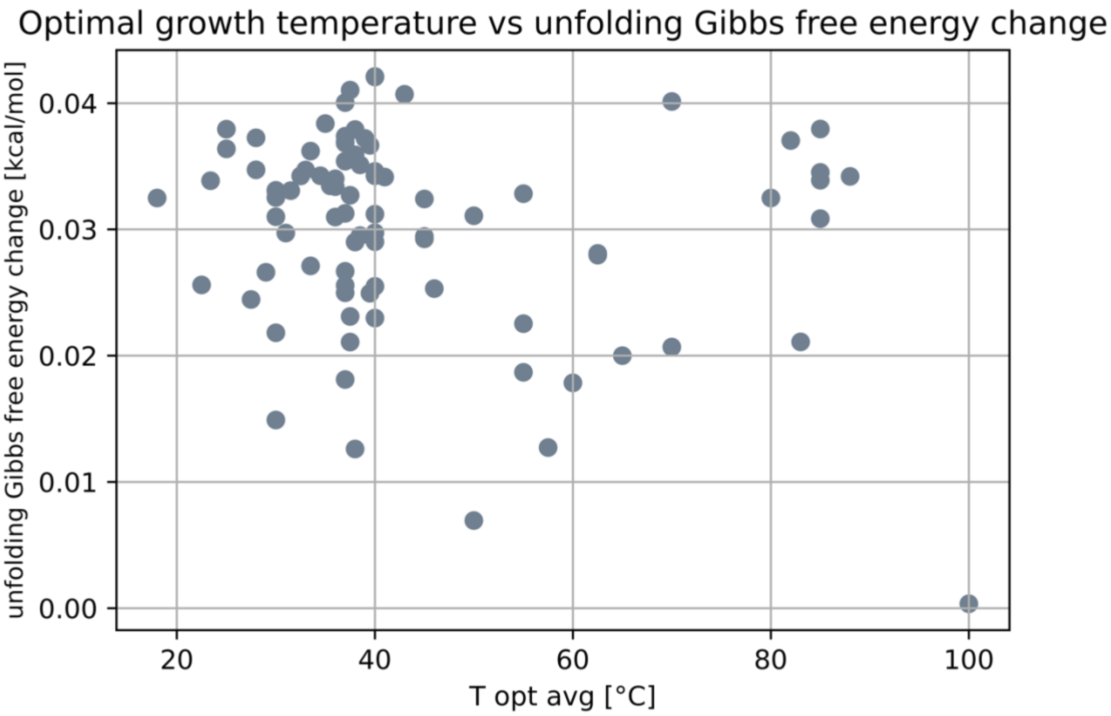
Optimal growth temperature vs unfolding Gibbs free energy change
unfolding Gibbs free energy change [kcal/mol]
0.00
0.01
0.02
0.03
0.04
20
40
60
80
100
T opt avg [°C]

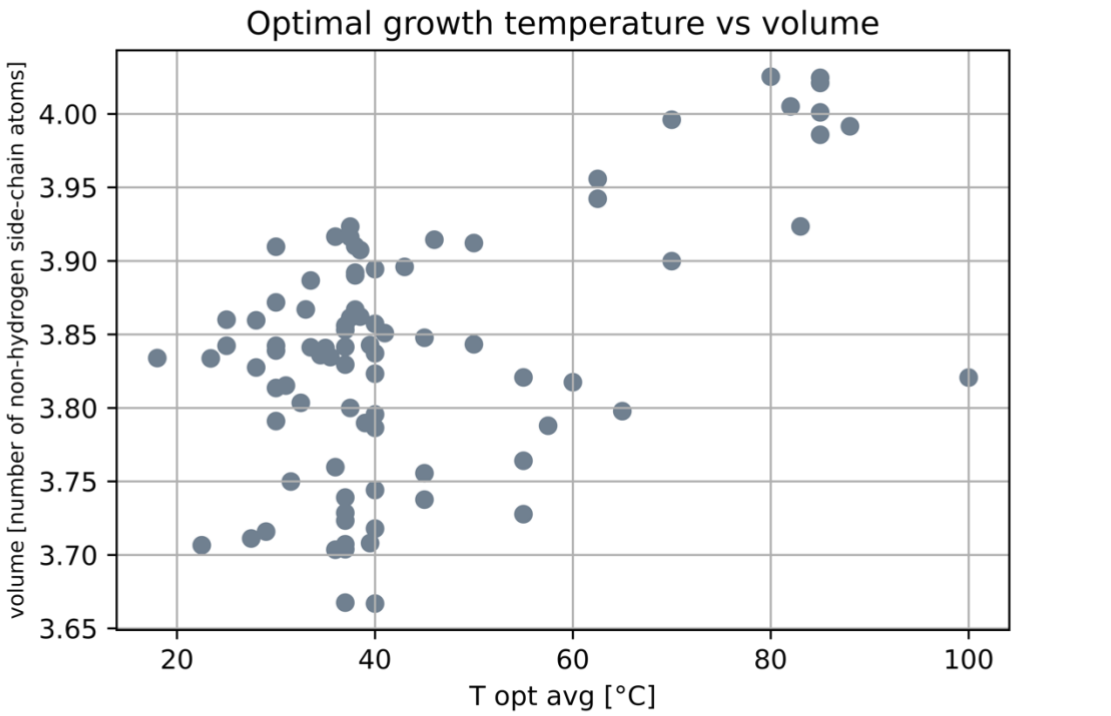
Optimal growth temperature vs volume
volume [number of non-hydrogen side-chain atoms]
3.65
3.70
3.75
3.80
3.85
3.90
3.95
4.00
20
40
60
80
100
T opt avg [°C]

Optimal growth temperature vs combined surrounding hydrophobicity globular and membrane

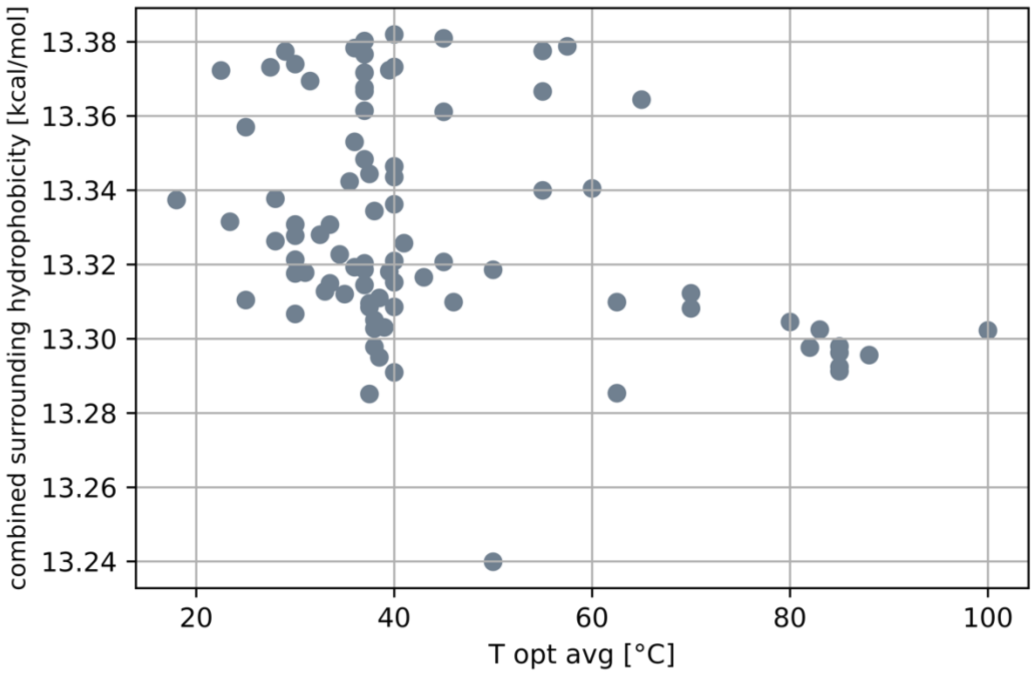

Optimal growth temperature vs solvent accessible surface area for denatured protein

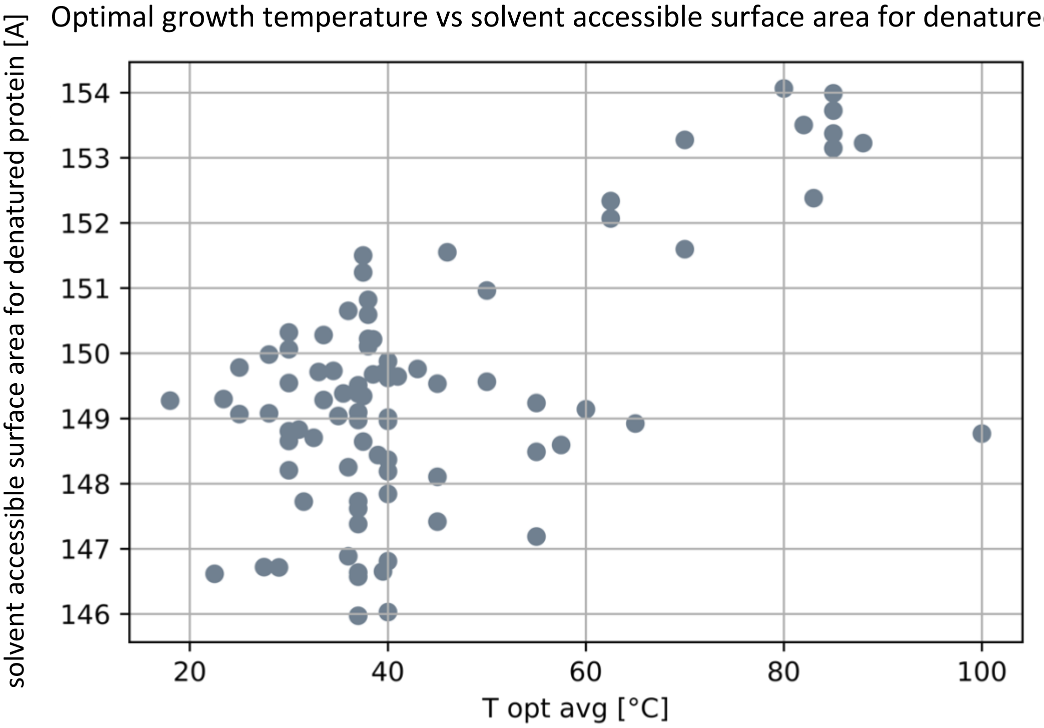

Optimal growth temperature vs solvent accessible surface area for native protein

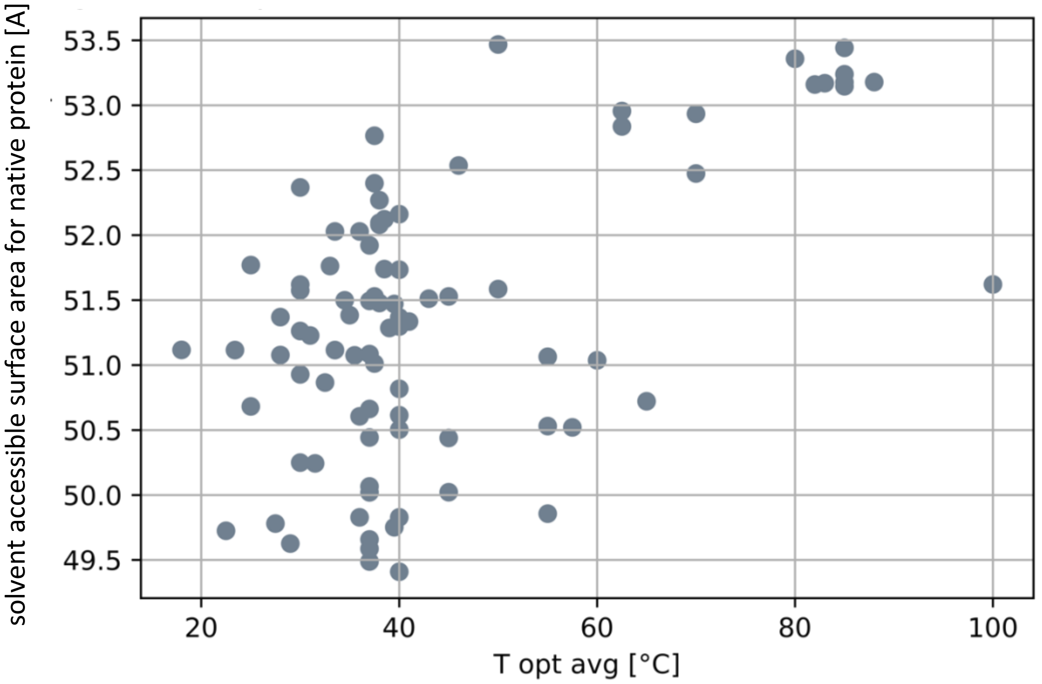

Optimal growth temperature vs thermodynamic transfer hydrophobicity

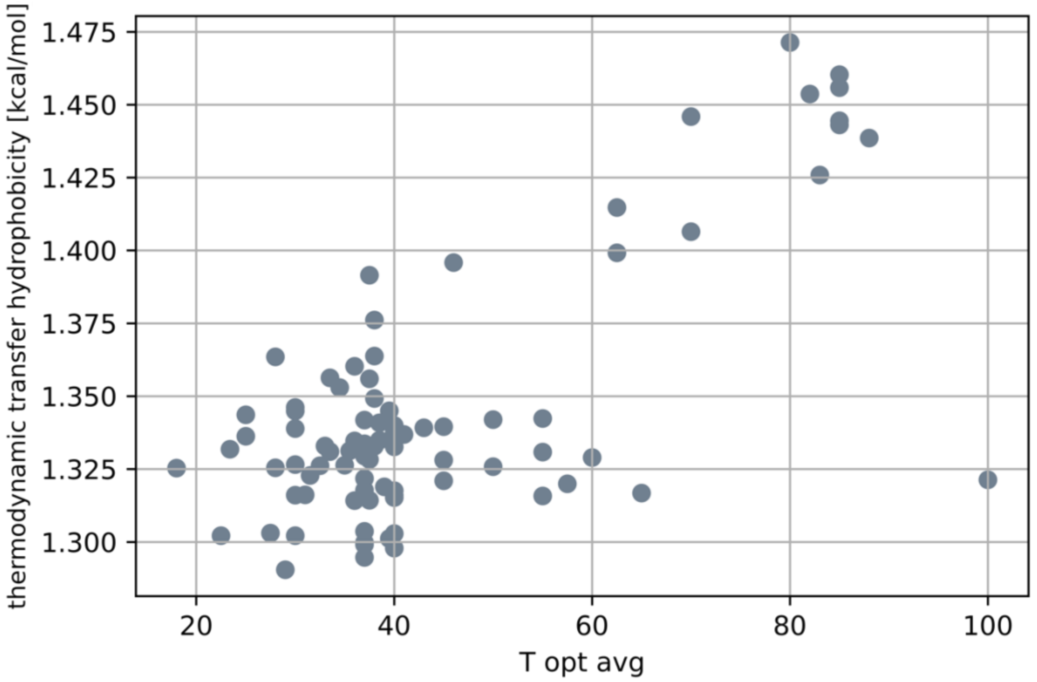

Optimal growth temperature vs short and medium range non bonded energy

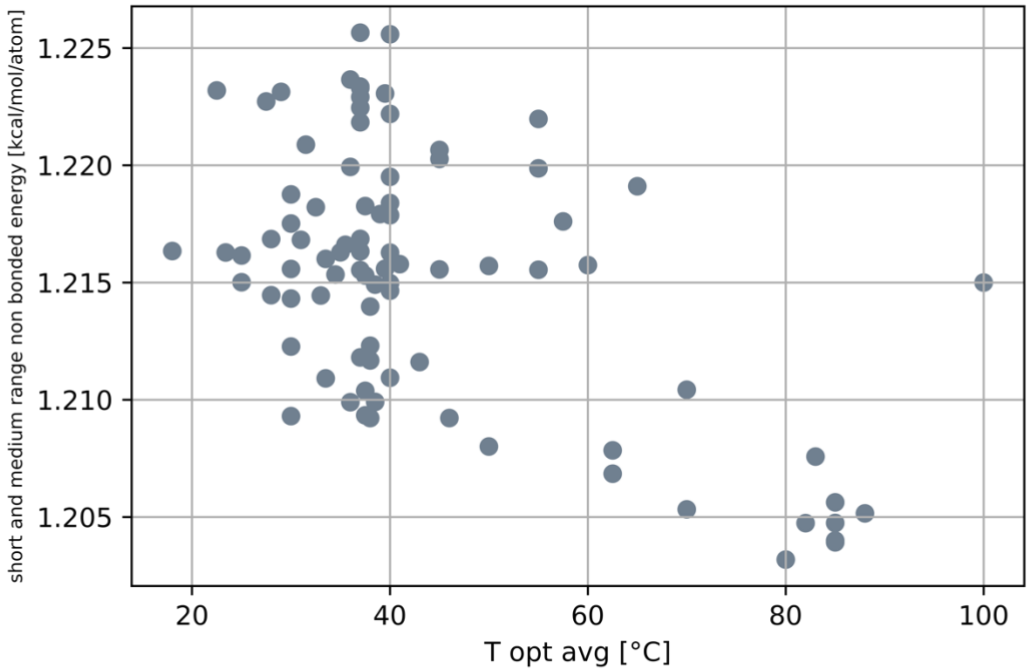

Optimal growth temperature vs unfolding heat capacity change

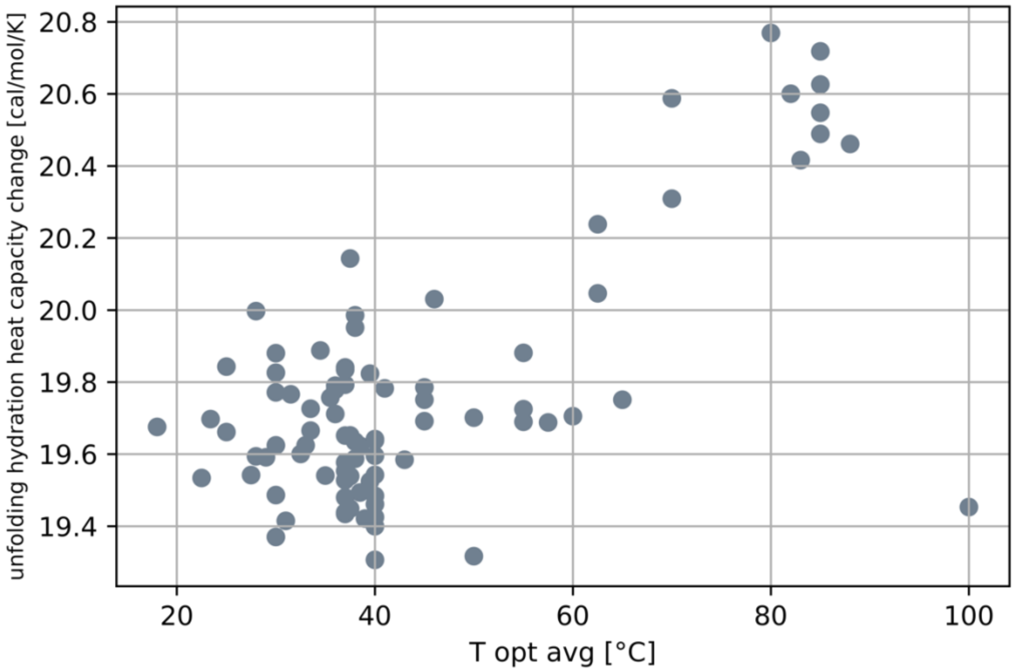

Figure S7: Gene duplication, gain and loss rates for each species and net gain rate with temperature.

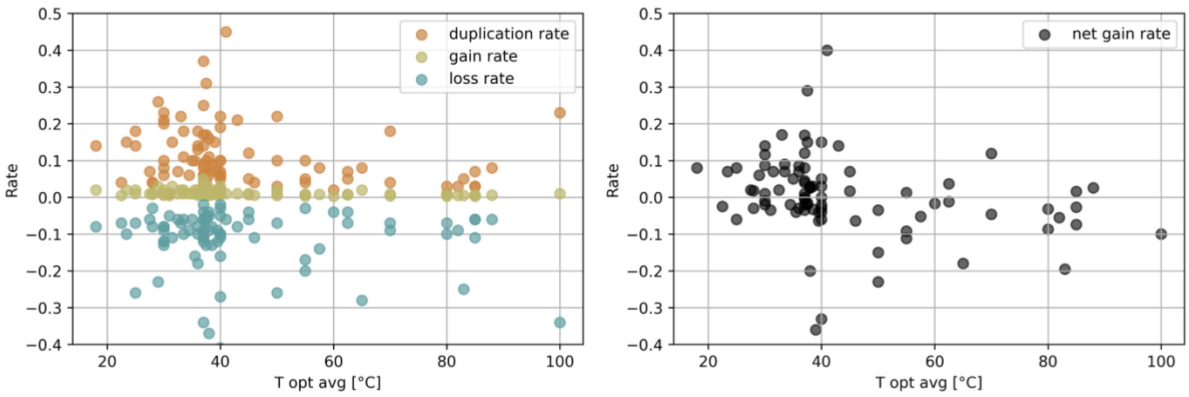

Supplement: dsac048_suppl_Supplementary_Figures [file dsac048_suppl_supplementary_figures.pdf]
